# Characterisation of Selected Materials in Medical Applications

**DOI:** 10.3390/polym14081526

**Published:** 2022-04-09

**Authors:** Kacper Kroczek, Paweł Turek, Damian Mazur, Jacek Szczygielski, Damian Filip, Robert Brodowski, Krzysztof Balawender, Łukasz Przeszłowski, Bogumił Lewandowski, Stanisław Orkisz, Artur Mazur, Grzegorz Budzik, Józef Cebulski, Mariusz Oleksy

**Affiliations:** 1 Doctoral School of Engineering and Technical Sciences, Rzeszow University of Technology, 35-959 Rzeszow, Poland; d554@stud.prz.edu.pl; 2 Faculty of Mechanical Engineering and Aeronautics, Rzeszow University of Technology, 35-959 Rzeszow, Poland; lprzeszl@prz.edu.pl (Ł.P.); gbudzik@prz.edu.pl (G.B.); 3 Faculty of Electrical and Computer Engineering, Rzeszow University of Technology, 35-959 Rzeszow, Poland; 4 Faculty of Medicine, University of Rzeszow, 35-959 Rzeszow, Poland; jacek.szczygielski@vp.pl (J.S.); kbalawender@ur.edu.pl (K.B.); boglewandowski@wp.pl (B.L.); sorkisz@ur.edu.pl (S.O.); drmazur@poczta.onet.pl (A.M.); 5 Department of Neurosurgery, Faculty of Medicine, Saarland University, 66123 Saarbrücken, Germany; 6 Institute of Medical Science, University of Rzeszow, 35-959 Rzeszow, Poland; damian.a.filip@gmail.com; 7 Department of Maxillofacial Surgery, Fryderyk Chopin Clinical Voivodeship Hospital No.1 in Rzeszow, 35-055 Rzeszow, Poland; robert.brodowski@wp.pl; 8 Institute of Physics, University of Rzeszow, 35-959 Rzeszow, Poland; jcebulski@ur.edu.pl; 9 Faculty of Chemistry, Rzeszow University of Technology, 35-959 Rzeszow, Poland; molek@prz.edu.pl

**Keywords:** guided bone regeneration, additive manufacturing, scaffolds, transitional implant, nanocomposite, hydroxyapatite, polylactide, 3D printing, cranioplasty

## Abstract

Tissue engineering is an interdisciplinary field of science that has developed very intensively in recent years. The first part of this review describes materials with medical and dental applications from the following groups: metals, polymers, ceramics, and composites. Both positive and negative sides of their application are presented from the point of view of medical application and mechanical properties. A variety of techniques for the manufacture of biomedical components are presented in this review. The main focus of this work is on additive manufacturing and 3D printing, as these modern techniques have been evaluated to be the best methods for the manufacture of medical and dental devices. The second part presents devices for skull bone reconstruction. The materials from which they are made and the possibilities offered by 3D printing in this field are also described. The last part concerns dental transitional implants (scaffolds) for guided bone regeneration, focusing on polylactide–hydroxyapatite nanocomposite due to its unique properties. This section summarises the current knowledge of scaffolds, focusing on the material, mechanical and biological requirements, the effects of these devices on the human body, and their great potential for applications.

## 1. Introduction

Functional and aesthetic problems related to tooth loss have accompanied mankind practically since time immemorial. Tooth loss is most often caused by trauma, infections, improper oral hygiene, or age-related factors. Dentures were manufactured and applied as early as around 700 BC. Initially, they were made from materials such as wood, bone, vulcanised rubber, or ivory. In the 20th century, materials such as polyvinyl chloride, vinyl acetate, cellulose plastics, modifications of Bakelite or poly(methyl methacrylate) were already in use. Currently, materials used in dentistry are divided into four main groups: metals, polymers, ceramics, and composites. They are used, among other things, for partial and complete dentures, permanent and temporary implants, denture linings, resin cements and fissure sealants. The materials used in the manufacture of dentures and implants should, above all, be biocompatible with the oral environment and mechanically resistant to the occlusal forces acting within the mouth [1].

The ideal materials for use in dentistry should meet several requirements that can be categorised. Chemically, they should not dissolve in fluids present in the mouth or ingested by the patient, nor should they absorb such fluids, as this causes dimensional changes. Furthermore, they are required to adhere very well to artificial teeth and liners. Biological aspects require that the material used be non-toxic, non-irritating, non-carcinogenic, and biocompatible [2]. A definite positive effect among some of these substances is their ability to inhibit the growth of bacteria responsible for caries, which forma biofilm on the teeth and cause infections [3]. In mechanical terms, the modulus of elasticity should be high, so that the relevant components, such as the denture base, will be rigid in relation to the acting occlusal forces. Furthermore, the resilience should also be high, so that the soft tissues underneath the placed component will be protected by absorption of chewing forces. High proportionality and elasticity limits prevent permanent deformation under occurring loads. For materials for dental applications, the specific gravity should be low and they are required to be dimensionally stable, while possessing sufficiently high abrasion resistance and sufficient mechanical strength to prevent cracking of the components under repeated biting forces [1]. The thermal expectations placed on the materials include their being good thermal conductors, their coefficient of thermal expansion being compatible with that of the teeth, and their softening point being higher than the boiling point of water. In terms of aesthetics, it is required that it is possible to dye or pigment the material and that the finished component should exhibit a level of translucency sufficient to match the oral tissues [2]. Among the factors that an ideal material for use in dentistry should fulfil that are not listed in the above categories are the retention of the desired properties for an appropriate period after manufacture, as well as a sufficiently long shelf life after application to the patient. Such materials should be relatively inexpensive and easy to work with, and sufficiently easy to clean and possibly repair. A final requirement is that they should be radio opaque, so that if an item is swallowed, it can be detected [3].

The use of biomaterials in dental implant therapy is becoming increasingly popular. Some of their characteristics have led them to become more readily used. Examples of such properties are: biocompatibility, bioresorbability, the ability to distribute drugs, and the ability to osteoconduction, which is particularly important because it stimulates the body to synthesise new bone. Mesenchymal stem cells first differentiate into cartilage cells and then into bone-forming cells [4]. The use of transitional implants actively drives the physiological process of differentiation into bone-forming cells, as they provide a three-dimensional framework that allows capillaries and perivascular tissue to grow into the graft. Host blood vessels enter the graft, leading osteoclasts to resorb the implant surface. As a result of the osteoconductive process, bone formations form on the implant surface, which ultimately leads to resorption of the original graft tissue, which is replaced by new host bone. This is a positive phenomenon, as the implant undergoes osteointegration, i.e., the fusion of the host tissue with its surface, which makes the use of resorbable transitional implants a better approach, as it does not require the removal of the implant fused with the bone [5].

Cranioplasty is a procedure that is widely used throughout the world. In this method, patient-specific implants are used to replace missing parts of the skull. Usually, such missing parts occur due to the removal of diseased anatomy or as a result of a decompressed craniectomy procedure used to relieve pressure on the swollen brain. The design of cranioplasty plates is becoming increasingly challenging. Computer-aided technologies and additive manufacturing techniques are being used to improve accuracy, increase accessibility and improve patient fit in the manufacture of such component [6].

**Figure 1:**
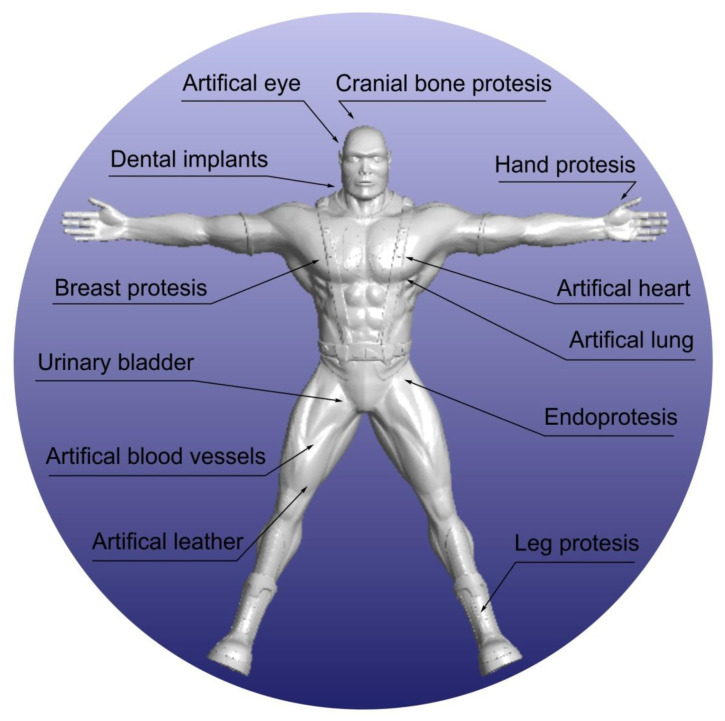
Examples of applications of implants and medical devices in the human body.

Initially, a variety of techniques were used for medical applications, such as foaming, pressing, casting and many others. Each of these had both advantages and disadvantages. However, these techniques were expensive or time-consuming, or the products were not adapted to the patient. Despite their drawbacks, these techniques are still used today for the manufacture of biomedical and ancillary equipment, as well as for some implants, abutments, and bone screws, among other things. Today, the dedicated techniques for making implants methods based on incremental manufacturing. Additive manufacturing has become, in recent years, a rapidly growing field, which enables both a more economical approach to production, while also making it possible to produce much more structurally complex parts, as opposed to the subtractive manufacturing methods used previously [7]. One of the most important additive manufacturing techniques is 3D printing. As a result of the expiry of patents, these techniques have undergone rapid development, thanks to which they are constantly being improved [8]. Thanks to modern computer-aided techniques such as CAD/CAM, it is possible to manufacture devices based on 3D scans, which is highly desirable in the context of medical and dental applications, as it enables decisive progress in personalised medicine (Figure 1). This not only makes it possible to make components work better through perfect fit, but also to avoid human error [9].

The authors decided to conduct this review because of the high degree of interest in the topic and the plans to develop the field. The interdisciplinary team unanimously believes that this topic has great potential for development and plans to carry out research work in this area in the future. One of the most important factors is the possible development of medical and dental techniques, which may have a direct impact on the quality of the procedures performed and a better effect on restoring the patients’ condition.

## 2. Materials Used in Dentistry and Medicine

### 2.1. Metals

Metals and their alloys play a major role in dentistry, particularly in the manufacture of implant restorations and conventional prostheses. On the one hand, they are unattractive as biomaterials because, as materials unnatural to the organism, they have no biological functions, are not biodegradable, and some of them show undesirable effects, including cytotoxicity. However, they do possess several properties thanks to which they are used in dentistry. Undoubtedly, their advantages include high strength, resistance to fracture and cracking, high workability, ductility, and electrical conductivity, as well as a good balance between stiffness and elasticity. Some of the most commonly used metals in dentistry are titanium and its alloys—Ti-6Al-4V, Ti-6Al-7NB, stainless steel, zirconium oxides, cobalt and chromium alloys (cobalt chrome)—and alloys based on precious metals—mainly silver and gold alloys. As the substances applied to the body must not be toxic, the metals used as biomaterials must be resistant to corrosion, as corrosion results in the dissolution of metal ions, so that they may exhibit toxic effects. The types of metals and alloys used usually have a passivating coating made of their oxides [7,10,11].

Titanium and its alloys are favoured as metallic materials used in both medicine and dentistry. They are used to manufacture dental implants, plates for maxillary prostheses, bridge wires, dental restorations, denture bases and bone screws [11]. They are characterised by high specific strength (strength to weight ratio), and their Young’s modulus is half that of stainless steel and cobalt chrome. Titanium is also opaque to X-rays and non-ferromagnetic, which means that patients with implants made from this material can be radiographed and can undergo magnetic resonance imaging. These characteristics make titanium and its alloys the preferred materials for medical applications, for example in bone abutments [12]. Due to the formation of stable titanium oxide layers on their surface, they exhibit good corrosion resistance, which is higher than cobalt–chromium alloys and stainless steel. Thanks to this, as well as good tissue compatibility, chemical stability and the osteointegration process, they can be safely used in the human body. The disadvantage of this metal is its low torsional strength (rotational modulus). The most conventional titanium alloy for medical applications is Ti-6Al-4V. It exhibits adequate machinability, heat treatability, weldability, as well as corrosion resistance, strength, and biocompatibility. Its yield strength is 895 MPa, which significantly impedes plastic deformation even under high load [12,13]. Suitable nickel-titanium alloys (equal atomic amounts in the alloy) exhibit exceptional mechanical properties—shape memory and superelasticity. The first of these properties is the ability to regain its original shape after deformation due to heating. Superelasticity is the property that makes it possible for all apparent plastic deformations to return to their original shape by releasing the load [14].

Magnesium and its alloys are used in various branches of medicine due to their suitable properties. Such materials have the potential to be used in biomedical applications as they have mechanical properties more similar to those of bone compared to titanium or steel, and are biocompatible [15,16]. They find applications as bone and compression screws, implants, and stents, among others. The products resulting from the degradation of this metal are mainly soluble magnesium ions, hydroxide ions and hydrogen gas. The unquestionable advantage besides the low price of the material is that these products are needed in the body or well tolerated by it, and their excess can to some extent be excreted with urine [17]. The natural occurrence of this metal and its potential to degrade are the subject of much research aimed at producing an implant that replaces natural tissue, but too rapid a corrosion rate does not leave enough time for tissue regeneration [18,19]. Although corrosion is a negative phenomenon, in the case of magnesium and its alloys it allows for antimicrobial activity [20]. Despite the undoubted advantages, the use of magnesium and its alloys as implants is limited by its disadvantages. In biological environments, it undergoes pitting corrosion in a relatively short time, which negatively affects the mechanical properties of the implant and may lead to its failure [21]. Moreover, during excessive corrosion, the formed products have negative effects on the organism. In the case of the previously mentioned basic products formed during corrosion, each of them has a different negative effect. Excess magnesium ions cause disruption of biochemical processes and normal cellular activities. Excessive concentrations of magnesium cause hypermagnesaemia [22]. On the other hand, high concentrations of hydroxide anions cause a significant increase in pH value, which is associated with toxic effects [23]. In the case of excessive corrosion, a large amount of hydrogen gas is released which, although non-toxic and easily diffused, can lead to gas bubbles that can cause emphysema [24]. Some of the material disadvantages for both titanium and magnesium can be eliminated by combining the two metals into a single alloy. The titanium matrix would provide mechanical properties. It is also expected that such a combination would reduce stress shielding. On the other hand, the contribution of magnesium can provide selective biodegradation of the implant—pore formation on the surface and in the implant volume, while at the same time having a stable titanium matrix [25].

Shape memory alloys are also an interesting group of metals. The possibility of this phenomenon occurring is determined by mechanisms such as unidirectional shape memory effect, pseudo-elasticity, and bidirectional shape memory effect [26]. The unidirectional effect consists in the transformation of the parent phase of the desired shape induced by the deformation to the original shape as a result of heating to a temperature characteristic for the alloy. The pseudo-elasticity phenomenon is associated with a reversible change under external stress. In this case, the return to the original shape occurs during heating, while no change of shape occurs during cooling, which results in the shape of the high-temperature parent phase only being stored. The elastic deformation created under these conditions completely disappears when the component is relieved [26]. The bidirectional alloy shape memory effect involves the retention of shape memory of both the high-temperature parent phase and the low-temperature martensitic phase. As a result of the bidirectional shape memory effect, in a specific temperature range, transformations take place cyclically, causing reversible changes in the shape of the object without the involvement of external stress. As the martensitic transformation usually results in the formation of martensitic laths with different orientations during the cooling process, apart from the volume change there is usually no macroscopic shape change. The preferred orientation of the martensitic nuclei has the effect of limiting the variants of lath orientation, causing anisotropic macroscopic shape changes. Transformations causing shape changes can be repeated cyclically by cooling and reheating provided that no removal of martensitic nuclei occurs during reversible transformation to the parent phase or by high temperature annealing [26]. Shape memory alloys find a variety of applications, including in medicine. Their use as long-term implants in surgery and orthopaedics, through the application environment, requires appropriate selection of alloy composition, heating method and temperature range. Known medical applications of shape memory alloys to date include clamps for osteosynthesis and treatment of rib fractures, plates for osteosynthesis, arch wires in orthodontics, bone nails, Harrington rods and spacer sleeves in the treatment of spinal disorders, clamps for aneurysms and blood clot filters. The use of implants made of shape memory alloys makes it possible to streamline and simplify many operations, as well as offering the possibility of introducing new surgical techniques. The introduction of shape memory alloys has also improved the technical level of medical equipment. Examples include structural changes to the artificial heart or miniaturisation of dialysis pumps. Modern applications of shape memory alloys also include needles for locating breast tumours, guide wire cores, tensioners used, e.g., as implants for vein dilation as a special type of stent, surgical instruments and adaptive endoscopes with a shape that adapts to the anatomical features of the patient during surgery or examination [26].

With the development of technology, biomaterials used in dentistry have been modified with nanoparticles. They are used in materials for dental fillings, pulp covering agents, implants, orthodontic appliances, and prosthetic base materials. Among their metallic representatives are titanium, zirconium, silver, gold, zinc, and copper. The use of these nanoparticles in materials has improved their antibacterial, mechanical and regenerative properties [13]. The addition of nanoparticles makes it possible to increase compressive, shear and tensile strength (Ag, TiO, ZrO_2_, Au), exhibit antimicrobial and antibiofilm effects (Zn, Cu, Ag, Au), and reduce frictional force. Furthermore, through the use of nanomaterials, implants can be developed that are capable of releasing drugs while maintaining therapeutic requirements such as drug loading, dosage and release rate [27,28]. Metal nanoparticles used in dentistry have a diameter of less than 100 nm, which increases their surface-to-volume ratio, biological activity, and chemical reactivity. Bactericidal activity is mainly due to the release of metal ions and the formation of reactive oxygen species able to react with the biological membranes of microorganisms, resulting in damage to their structure and inactivated bacteria [29].

Despite the many undoubted advantages and possibilities associated with the use of metallic materials, they unfortunately have several characteristics that limit their use in modern applications. Metals and their alloys currently allow the safe use of an implant for a period of about twenty years, and many of the applications, such as spinal treatment or endoprosthesis, require a longer period [30,31]. Implantology offers the possibility of replacing damaged anatomical structures and restoring their lost functions. This involves the introduction of foreign bodies into the human internal environment with the assumption that they will be biologically inert while fulfilling their designated function over the long term. Thus, the key issue related to the implantation of metallic materials is their susceptibility to individual toxicological and allergic reactions and tolerance to mechanical irritation [31,32]. Modern design and material solutions have reduced local and systemic complications. Despite this, problems associated with the development of inflammatory-degenerative changes, bone destruction and aseptic loosening are still present [32]. The long-term use of the implant in a body fluid environment places particularly high demands on metallic materials used in medicine with regard to high resistance to pitting corrosion, stress corrosion cracking, and the lowest possible corrosion current density. The risk of damage to the passive layer during surgery and in use requires that the metallic biomaterial be highly self-assimilating. Ensuring appropriate electrical properties of metallic biomaterials is also an important problem. Significantly lower resistivity specific for steel than for bone or muscle tissue causes disturbances in the regeneration processes of the bone tissue adjacent to the implant [30,31]. One of the problems resulting from the use of metallic materials in medicine is the high risk of post-operative complications and infections. This fact should be combined with adverse reactions of hydrogen release and oxygen uptake from tissues in the vicinity of the implant. The local decrease in pH and oxygen concentration damages the surrounding tissues, weakens the resistance to bacteria and increases corrosive processes [31,33]. An important issue is also the preparation of the metal implant surface in order to ensure an adequately durable and strong connection between the implant and the surrounding tissue without compromising the co-corrosion resistance and mechanical properties. Research has been conducted to obtain a permanent connection between the implant or cement and the bone tissue, which is subject to constant stress and metabolic processes [32]. The most common failure after total hip cement prostheses is the loosening of the prosthesis components. Too stiff stems of cementless endoprostheses carry most of the load that was previously carried only by the femur. This leads to increased resorption around the stem, weakening of the bone, and ultimately to perforation of the femoral cortex [31]. Despite these challenges, metal nanoparticles play a very important role in modern medicine. Their applications, advantages and disadvantages are summarised in Section 2.4.

### 2.2. Polymers

**Figure 2:**
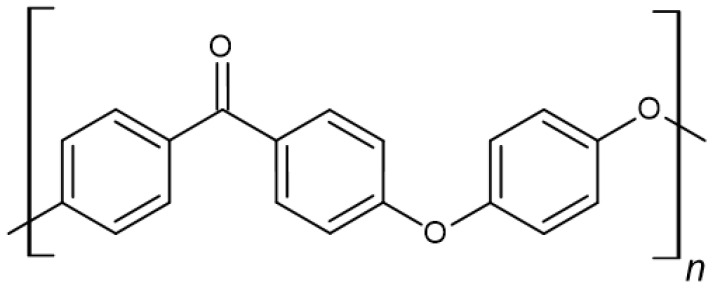
Polyether ether ketone structure.

**Figure 3:**
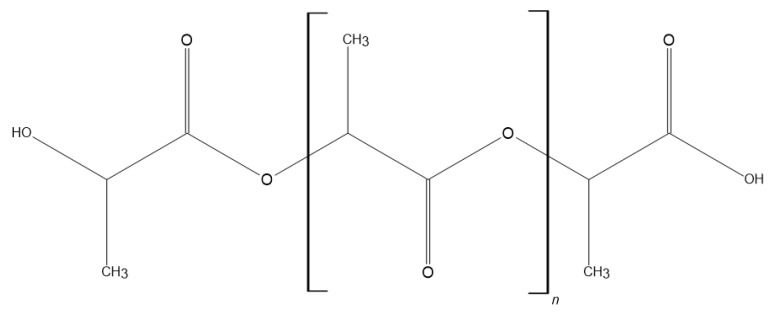
Polylactide structure.

Polymeric materials are increasingly being used in medical and dental applications, thanks to their display of desirable biological and mechanical properties, ease of processing, low cost of production, and the possibility of obtaining excellent surfaces for both polymeric materials and films. The above attributes allow them to be adapted to a wide range of applications. The most commonly used for medical applications are poly-L-lactic acid (PLLA), polyetheretherketone (PEEK) (Figure 2), polylactide (PLA) (Figure 3), and polymethylmethacrylate (PMMA). The use of polymeric materials in dentistry is becoming increasingly popular due to the characteristics they exhibit. Thanks to their antimicrobial properties, they are used as drug carriers and in regenerative, reconstructive, and prophylactic therapies. Polymeric materials are also used to reduce friction and corrosion, as dental adhesives and to regenerate tooth pulp and dentin. Bioactive polymers are used as advanced drug delivery systems. Polymer materials and polymer composites meet dental requirements such as mechanical and biological properties, corrosion behaviour, availability, cost, aesthetics, and relative ease of processing. In addition, the use of polymeric coatings enables increased biocompatibility of bulk materials [2].

The Table 1 shows the polymers most commonly used in medicine and dentistry.

Among the many polymeric materials with potential for use in dentistry, polyether ether ketone (PEEK) is one of the most promising. It is a thermoplastic polymer material with low specific gravity and high impact strength. It has physical and mechanical properties similar to those of bone. It is transparent to X-rays. It is used in many fixed and removable prosthetic restorations, healing screws, and is also used in the manufacture of aesthetic orthodontic wires due to its ability to exert more favourable forces compared to conventional wires [10]. The advantage of PEEK is that medical implants made from it can be manufactured using 3D printers. It is characterised by low solubility in water (0.5 w/w%) and does not undergo chemical changes even at high temperatures (up to 300 °C); thanks to this, it is a material that can be subjected to the process of sterilisation [37,38]. Due to its semi-crystalline structure, PEEK is less brittle than crystalline materials. Its modulus of elasticity (3.1 GPa) is similar to that of bone, which is an unquestionable advantage, as it reduces the stress transferred to the abutment teeth and cement connections. It is also easy to modify by adding other materials. Since PEEK has a lower tensile strength (80 MPa) and Young’s modulus (3–4 GPa) than teeth or dentin, it is often used as a polymer matrix for composites. For example, the combination with carbon fibres (CFR-PEEK) allows a significant increase in these parameters—the tensile strength of the composite increases to 120 MPa, and its Young’s modulus to 18 GPa [39,40]. PEEK is characterised by abrasion resistance comparable to metal alloys and shows higher abrasion resistance during lateral forces. The polymer is classified as a biocompatible material, and there is no evidence to suggest potential carcinogenic, mutagenic, cytotoxic or immunogenic effects [41,42]. Although it is a relatively bioactive material, its binding to bone is limited. To improve this phenomenon, PEEK composites with hydroxyapatite, coating of PEEK with hydroxyapatite and titanium, the creation of network structures allowing tissue ingrowth, including phosphate–calcium biomaterials, and other modifications are being developed. However, the dental use of PEEK is limited at this time, as it requires long-term clinical studies [10,43].

Another widely used biopolymer is polylactide (PLA). This biodegradable and thermoplastic polymer can be easily synthesised from renewable resources. PLA is a biocompatible material used in regenerative medicine, tissue engineering, implant manufacturing, as a drug carrier, in skin and tendon healing processes. Polylactide is approved by the FDA (Food and Drug Administration) as a material for direct contact with biological fluids. It is also used in the manufacture of medical instruments and equipment. It is a three-dimensionally printable biopolymer, which is its undoubted advantage [44]. Thanks to PLA’s properties, it can be used for rapid prototyping, and by using it in 3D printers, it is possible to generate the necessary patient-specific components. It is possible to customise the mechanical properties of this polymer. This is because the lactide monomers are chiral, so that by appropriate polymerisation of L-lactide, D-lactide, D,L-lactide or mesolactide, it is possible to manipulate the properties of the resulting PLA.To improve the slowing down of the degradation rate, to obtain better thermal stability, or to increase hydrophilicity, PLA can be blended with other polymers, including polyethylene, polystyrene, polypropylene or polyethylene glycol. In the case of polylactide, the degradationrate can also be adjusted, which depends on several factors: polymer composition, its molecular weight, crystallinity, pH, additives, production processing, mechanical stress, type of sterilisation and geometry of the manufactured part [45,46]. Controlling the rate of degradation of PLA and its composites is beneficial during medical surgeries, as it reduces the number of surgical interventions for the patient; moreover, it can be used for controlled-release drug carriers and transitional implants. The use of this polymer as a drug carrier allows it to be carried across biological barriers such as the blood–brain barrier. This makes it possible to apply the drug bypassing the metabolic processes affecting the therapeutic substance administered without the carrier. This allows the development of targeted therapies—the drug is released in the right place, making it much more effective—such as, among others, cancer therapies. The use of carriers also allows the use of therapeutic substances with low molecular weights without the risk of them being filtered out of the blood before reaching the right place. PLA as a carrier material for drugs is used during implantation procedures as it reduces the risk of post-operative infection, thereby reducing the likelihood of failure [47,48]. Polylactide has excellent bioresorption capabilities, whereby the polymer shows the ability to integrate with host cells and tissues; however, due to the hydrophobic nature of PLA, proteins and cells show limited surface interaction, the promotion of cell penetration is reduced, and there is the potential to induce an inflammatory response. Due to the biological and mechanical properties of polylactide, it is not used as a pure unfilled and unmodified material [49]. However, in the case of manufactured composites, its properties are significantly improved, which makes it possible to utilise its strengths by compensating for the weaknesses of this material. When combined with hydroxyapatite (HA), the flexural strength is increased, and the material also shows the ability to stimulate osteogenesis by activating pre-osteoblastic cells and osteoblasts. This combination increases the roughness of the material while reducing its wetting angle. This allows for increased protein adsorption and interaction of the material with the extracellular environment. The increased wettability improves the hydrophilicity of the material and the rough topography increases pre-osteoblast proliferation and differentiation, which is beneficial for bone growth [49,50]. Polylactide is used in dentistry due to its structural adaptability and biocompatibility. The undoubted advantage is also the possibility of drug delivery, thanks to which there is both protection against infection and prevention of the appearance of inflammation in the case of implantology. It is able to effectively stimulate osteointegration of dental implants with native hard tissue in the oral cavity. A properly prepared composite containing PLA can be used as a transitional implant, as it is able to allow bone regeneration in the oral cavity. In dentistry, many restorative processes are based on resins, which significantly improve their mechanical properties when combined with polylactide [51]. The PLA composite scaffolds in the resin result in increased flexural strength, modulus and compressive strength in the material compared to conventional resins. Membranes made by combining polylactide with other polymers allow for increased oral bone regeneration, which has been studied using rats. Membranes made from a PLA-PGA (polylactide–polyglycolic acid) copolymer have been studied. Thanks to many properties of great value to medicine and dentistry, polylactide is a widely used biomaterial [51,52].

Another type of these compounds is thermosensitive polymers, otherwise known as intelligent materials—depending on the ambient temperature, they change their solubility, which is accompanied by a conformational change in the polymer structure. Thanks to their ability to react to temperature changes, they find several biomedical applications such as tissue engineering, drug delivery, separation and recovery of cultured cells or nanomedicine. They can also be fabricated using 3D printing. In the case of thermosensitive polymers, they make it possible to induce, under the influence of temperature change, changes in the physical properties of the material, such as gelation (topical applications and biodegradable scaffolds for injection), stimulated swelling, and collapse of hydrogels, causing a change in surface properties (in vitro applications in cell culture) [53]. The ability of a material to respond to stimuli allows the properties of the material to be controlled in some way by environmental conditions. What is important about these polymers is that some of them do not require temperatures outside the range that is safe for the human body to act thermoreactively. One example of such compounds is poly-N-isopropylacrylamide (PNI-PAM) and its derivatives. Thermosensitive polymers can be divided into two groups: type LCST (lower critical solution temperature) and UCTS (upper critical solution temperature) [54]. LCST-type polymers are completely miscible in a solvent with a temperature lower than the transition temperature. Phase separation occurs above this temperature—the polymer then changes its conformation to a coiled, more slender form. This change in solubility occurs because it is more energetically favourable. Below LCTS, solubility occurs because of hydrogen bonding interactions with surrounding water molecules and because of limited intramolecular and intermolecular hydrogen bonds. Polymers of this type exhibit rapid, sharp and reversible phase transitions in response to temperature change as it leads to a change in the entropy of dissolution of polymer chains in aqueous solution. UCTS polymers present the opposite mode of action—they exhibit phase separation upon cooling. It is also possible to control the transition temperature in their case by changing the copolymer composition, chemical structure, or different contents of amino groups. Unfortunately, polymers with UCTS do not show biomedical properties as good as those of LCTS. Thermosensitive polymers have great potential in biomedical applications. The possibility of delivering drugs while taking into account the circadian rhythm of the patient or the progress of gene therapies or tissue engineering show that these are very promising materials [55].

#### Application of Polymeric Materials in Orthopaedics

**Figure 4:**
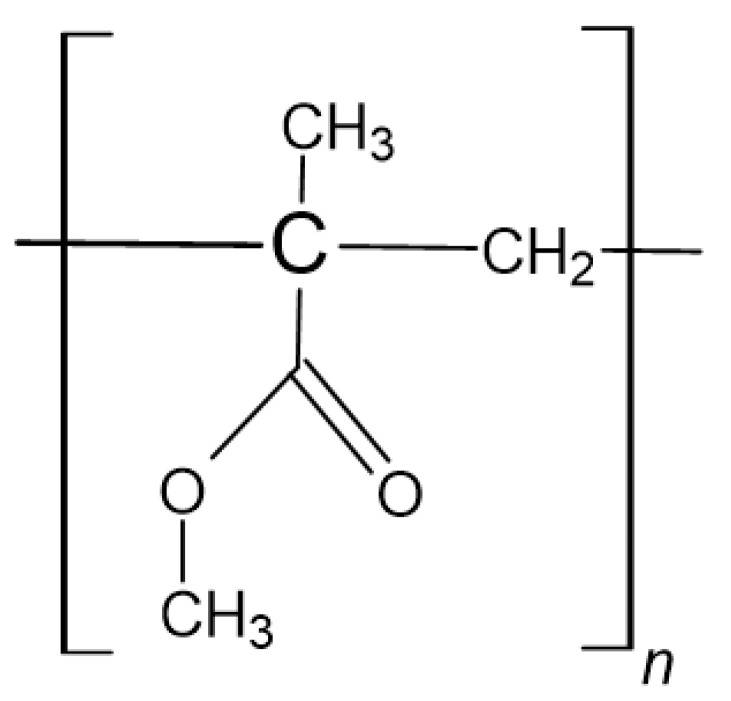
Poly (methyl methacrylate) structure.

Globally, the number of injuries is increasing, as is the number of complex and costly surgical procedures. The need to develop effective and reliable materials for the rapid and uncomplicated tissues regeneration is undeniable. In orthopaedics, this all started in the early 1960s, thanks to the English surgeon Sir John Charnley and his research on bonding prostheses to bone using poly(methylmethacrylate) (PMMA) (Figure 4). Since then, polymers have gained increasing importance in orthopaedic surgery. For the last nearly 60 years, many in vitro and in vivo trials have been carried out with newly created polymers, and some of them are now being successfully used. By searching only one PubMed database, it can be concluded that this topic is still very relevant. Generally, the polymers currently used in orthopaedics can be categorised into two groups: absorbable versus non absorbable [56].

PMMA-based non-absorbable bone cements are one of the most abiding and widely used materials in orthopaedic surgery. Due to their properties for fixing implants to the bone, they are used in total joint replacement. They are also used in tumour surgery and in percutaneous vertebroplasty. Acrylics can act as a temporary cement spacer to manage a post-traumatic bone defect and in two-stage joint revision arthroplasty due to infection. By mixing the ground polymer with monomer, a dough is obtained that can be manipulated and moulded. During the working phase, cement must be liquid enough to penetrate the interstices of cancellous bone, achieving micro-interlocking. PMMA-based bone cements are produce in varying viscosities: low, medium and high [57].To improve bioactivity, biocompatibility, osteointegration ability, and some other properties, bioactive additives are used to modify PMMA bone cement [56]. For example, quite promising are studies in which hydroxyapatite-modified PMMA bone cements (HAP-modified PMMA) exhibited longer setting times, lower maximum exothermic temperatures while curing, and higher mechanical properties [56,58]. Since the 1970s, various antibiotics have been added to PMMA-based cements to reduce the adhesion of bacteria to the bone cement surface and for their effective local release [59,60]. Because there are still doubts about antibiotics amount, their release, and the mechanical performance of antibiotic-loaded bone cements, a lot of effort has been directed toward developing antibiotic-free bone cements with antibacterial properties [59,60,61,62]. Additives with antibacterial properties have become a hot topic of research, but none of them is perfect [62,63,64,65,66,67]. Among them, the synergistic properties of mixtures formulated from both chitosan and graphene with PMMA, suggest that they possess very high potential to be used as antibacterial bioactive cement in orthopaedic applications [65]. To overcome the weaknesses of current antibiotic bone cements, titanium dioxide nanotubes (TNT) were bonded to PMMA bone cement containing antibiotics (vancomycin or gentamicin). In this combination, the mechanical properties of the cement were well pre-served, but more than 50% of the antibiotic was released, instead of only about 5% in the absence of TNT within 2 months [68]. The combination of a bioactive and an inorganic antibacterial agent with PMMA bone cement seems to be an efficient and attractive alternative to PMMA-based bone cements alone, since it simultaneously allows a better bond with bone and stronger limiting of bacterial adhesion and proliferation [59].

After PMMA, the most commonly used polymer is polyethylene (PE). It is still the gold standard for bearing surfaces in total joint arthroplasty. To increase arthroplasty longevity and improve wear resistance, new types of PE have been designed [69,70]. Commonly used types includethe first-generation highly cross-linked polyethylene (HXLPE) and the second-generation vitamin E-stabilised HXLPE (E1). HXLPE-based products have been used not only in joint arthroplasty but also for fabrication of arthroscopic tools and implants [71,72,73,74,75,76].

Polyetheretherketone (PEEK) and its forms, members of the polyaryletherketone (PAEK) family, are well-known nonabsorbable polymers with excellent chemical and thermal resistance, biocompatibility, corrosion resistance, X-ray radiolucency, sterilisation performance, mechanical wear characteristics, and stability. To increase their biological properties, surface modifications of the PEEK and PEEK composites have been proposed [77]. PEEK polymer is mainly used in spinal implants, including spinal fusion cages to stabilise the anterior column of the lumbar or cervical spine, disc arthroplasty, and as interspinal spacers, but also in foot and ankle fusion. PEEK anchors and interference screws have many advantages over metal and bioabsorbable ones during arthroscopic procedures, e.g., ACL reconstruction and meniscal repair [38,77,78].

Polypropylene is another non-degradable polymer, with characteristic properties including low density, relatively high thermal stability, easy processing, and resistance to corrosion. It is mainly used for the fabrication of ultra-high-strength rods for bone fixation and some spinal stabilisation systems. Additives such as boron nitride, nHA, and the linear polymer chain of carbon atoms improve its biocompatibility and mechanical strength [79].

**Figure 5:**
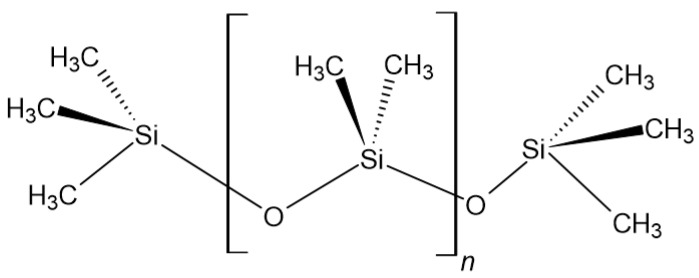
Polydimethylsiloxane structure.

Polydimethylsiloxane (PDMS) (Figure 5), commonly known as polysiloxane or silicone, has good flexibility, is a chemically stable material, resistant to extreme temperatures, aging, oxidation, and moisture. The most common application of PDMS in orthopaedics is in hand and foot surgery, including interphalangeal and metacarpophalangeal implants and radial head implants [80].

Implants composed of absorbable polymers (natural and synthetic) have several advantages over metallic and nonabsorbable ones. These include a reduction in stress shielding and the resulting bone weakening, and there is usually no need to remove them. In addition, they can act as a structural support and delivery devices for various substances, e.g., antibiotics or growth factors, which are gradually released as the implant degrades. The disadvantages include lower strength, higher cost, and in some cases the induction of a non-specific local inflammatory reaction. To increase the mechanical strength, antibacterial properties, and osteoconductivity, and to decrease the incidence of adverse tissue reactions, various materials can be added, e.g., hydroxyapatite, calcium phosphate, ceramics or bioactive glass. Natural biodegradable polymeric biomaterials generally include proteins (collagen, fibrin, etc.), and polysaccharides (starch, alginate, hyaluronic acid derivatives, etc). The most often used biodegradable synthetic polymers are Polylactide (PLA), Polyglycolide (PGA), or a combination of the two and Poly(ε-caprolactone) (PCL). Bioabsorbable polymers in orthopaedics are well known for applications in bone repair and regeneration, in sport medicine, in trauma surgery (degradable sutures, plates, pins, nails and screws), and as tissue engineering scaffolds [60,81,82,83,84,85,86,87].

Summarising the topic of the use of polymers in orthopaedic surgery, it should be noted that both degradable and nondegradable groups positively impacted the development of the field of orthopaedics, not only in the production of implants, drug release systems, and tissue engineering (tissue replacement, repair, and regeneration), but also in orthopaedic research and education.

### 2.3. Ceramic Materials

The most commonly used ceramic biomaterials in dentistry are made from calcium phosphates, halloysite, alumina, and zirconia. Ceramic materials have been known and used in medicine for many years. Compared to other biomaterials, ceramic biomaterials are characterised by porosity that allows tissue ingrowth and secures a permanent connection between tissue and implant, high compressive strength and abrasion resistance, high corrosion resistance in the tissue environment, the possibility of sterilisation without changing the material properties, and brittleness [88]. Some human tissues such as bones or teeth consist mostly of solid inorganic material (70–97% hydroxyapatite), so ceramic materials may be more effective as implants than the metals or plastics used so far. This is particularly true for hydroxyapatite bioceramics, which have the same chemical and phase composition as human bone. Ceramic biomaterials, apart from their many advantages, have significant disadvantages—they are brittle materials with low flexural strength, are non-deformable, not are resistant to dynamic loads [89,90]. The flexural strength of alumina bioceramics is 400 MPa and that of hydroxyapatite 150 MPa, while for human bone this value does not exceed 120 MPa [31,91]. Despite higher values than those for human bone, the flexural strength of ceramic biomaterials is not sufficient. This is due to the delayed failure phenomenon occurring in ceramic biomaterials related to the growth of subcritical cracks. This phenomenon means that even a positive result of the strength test does not give a guarantee that under operating conditions, catastrophic failure will not occur when carrying loads much lower than critical [31]. This property affects the limitation of the area of application in medicine of biomaterials made only of bioceramics. Ceramic materials are not resistant to dynamic loads and do not exhibit deformability. High hardness and good resistance to abrasion and corrosion in tissue and body fluid environments minimise, but do not completely eliminate, the wear of ceramic biomaterials after long-term use. The wear products of this group of materials do not cause significant toxic and allergic reactions, which determines the good biotolerance of bioceramics in the body [30]. Each material is capable of producing a peri-implant reaction. Connections at the implant boundary between bioceramics and tissue depend on the reactions occurring there. The types of connections between bioceramics and tissue can be divided into the following groups: when the material is toxic, the tissue dies; when the material is non-toxic and biologically inactive, a fibrous tissue of variable thickness is formed; when the material is non-toxic and biologically active, the separation surface is chemically bound; and when the material is non-toxic and resorbable, it is absorbed into the surrounding tissues [92]. The main medical applications of ceramics can be divided into two groups. Porous ceramics are used for mandibular prostheses, artificial bone segments and permanently fixed artificial limbs. Non-porous ceramics are mainly used in the manufacture of joint endoprosthesis components. In addition, ceramics are used for prosthetics of the auditory bones, reconstruction of nasal and orbital bones, artificial tooth roots, tracheal rings, filling of bone defects and as a root canal sealant.

Calcium phosphates are one of the most important groups used in dentistry. They can induce the repair and reconstruction of bone defects. An important reason for their use is their similarity to the inorganic fraction of mineralised tissues such as bone, dentin, enamel, or cementum, especially tooth enamel, which consists of 98% by mass of large crystals of biological apatite. Of the calcium phosphate group, hydroxyapatite (HA)(Ca_10_(PO_4_)_6_(OH)_2_) is the most commonly used in dentistry, but fluorapatite (FA) (Ca_10_(PO_4_)_6_F_2_) and hydroxyfluorapatite (HFA) (Ca_10_(PO_4_)_6_(OH_x_F_y_)) are also used [93]. Due to their poor mechanical properties, they are rarely used as stand-alone materials; however, due to their biological and structural properties, they are widely used as composite components. Due to their high level of biocompatibility and bioactivity, as well as the fact that they do not exhibit toxic or allergenic properties, they are used as elements of composite materials, bone substitutes, bone cements or implant coatings. Studies show better biological properties for FA or FHA than for HA [94,95]. Better proliferation and fewer dead cells have been observed on the surface of fluorine-containing apatites. The main task of conservative dentistry is to maintain the natural dentition in a healthy state by means of preventive treatments, strengthening tooth tissue and preventing changes associated with certain diseases such as caries or hypersensitivity. However, in cases where such treatments are insufficient, invasive methods are used [96]. HA is then used as a material or component in the production of implants, scaffolds, blocks, implant coatings or cements. It has a positive effect on creating a specific bond between the bone tissue surrounding the defect and the implant (osteointegration) and on inducing bone cell growth and development (osteoconduction). As a result, the bone tissue in the defect area regenerates faster, greater implant stability is ensured, the risk of bacterial infection is reduced, and the risk of the implant being rejected by the body is also reduced [97]. Hydroxyapatite also increases the durability of implants, as clinical studies have shown that an implant containing an HA coating has a significantly longer lifespan compared to implants without coatings. Coatings with FA show a greater extension of implant material life and better osteointegration properties compared to those with HA, due to the poor solubility of FA in acidic environments and its stability. Highly porous materials can be created using HA, resulting in faster resorption and higher osteoconductivity. As a result, they are used in bone defects for guided bone regeneration, for example, in situations of jaw bone atrophy, which most often occurs as a result of parodontosis or as a result of defects following tooth loss [98]. To prevent phenomena that make it impossible to insert an implant in the defect, such as lowering of the bottom of the maxillary sinus or protrusion of the upper jaw teeth corresponding to the defects in the lower jaw, transitional implants with HA in the form of granules or blocks are used, which will appropriately load the defect site. As a result, the bone, which forms the basis for the permanent implant, will be rebuilt. This is made possible by creating a composite membrane with an asymmetric pore distribution. One side consists of a spongy layer of macropores and the other of a dense layer of micropores. These prevent the migration of fibrous connective tissue, while at the same time permeating the components involved in bone regeneration. This material shows a high affinity for binding bone marrow stromal cells, while at the same time having no negative effect on cell proliferation [98,99]. The above characteristics indicate the appropriate biocompatibility of the element and the possibility of application in guided bone regeneration. Cements are also made from calcium phosphates, which are self-curing materials that solidify after application at the target site. The main advantages of cements with HA as the starting material are biocompatibility, fast setting time, osteoconductivity, ease of delivery to the target site, and good plasticity [93,95,98].

Zirconia ceramics are also widely used in dentistry. This material has exceptional mechanical properties and is easy to process in the pre-sintering phase using CAD/CAM. Thanks to its osteoconductive effect and biocompatibility with oral tissues, it is used to manufacture dental implants, abutments, dental bridges, crowns, finials, and orthodontic brackets [100,101]. Zirconia ceramics are tasteless and do not cause allergic reactions. In addition, it is a material of high hardness, abrasion resistance, strength, and corrosion resistance. Its modulus of elasticity is similar to that of steel, its coefficient of thermal expansion is similar to that of iron, and its resistance to fracture is the highest among the most commonly used ceramic materials. To improve its mechanical properties, zirconia ceramics are stabilised by adding yttrium [102,103]. Stress-induced transformation from a tetragonal to a monoclinic form then occurs during fracture. The accompanying increase in volume creates a compression zone that shields the crack tip, thus inhibiting crack propagation and increasing the strength of the component. Despite its many advantages, zirconia ceramics also have disadvantages. Opacity can have a negative effect on the aesthetics of the finished part. The ageing process promoted by moisture facilitates degradation and an increase in surface roughness and the occurrence of cracks. These factors can result in a definite deterioration in performance after time [101,102,104].

Halloysite(Al_2_Si_2_O_5_(OH)_4_×*n*H_2_O) is an aluminosilicate mineral possessing a characteristic tubular morphology. It is used in bone tissue engineering, dental fillings, implants, tissue scaffolds, drug delivery and functional substances [105]. Halloysite nanocomposites are used in wound healing. The mineral is most commonly used as an enhancer to improve several properties of nanocomposites—corrosion resistance, mechanical properties, antimicrobial osteoconductivity, and cell adhesion and proliferation [106,107]. It has great potential for medical applications due to features such as large aspect ratio, unique tubular morphology, low cytotoxicity, distribution of different charges, high availability and low cost [108]. Halloysite has a positive internal charge and a negative charge on the surface. It is stable under alkaline and neutral conditions. Because of this, as well as its unique morphology and ease of modification with other materials, it is possible to use both modified and unmodified halloysite nanotubes (HNT) to encapsulate a wide variety of substances, positively charged, negatively charged or uncharged, as well as both hydrophilic and hydrophobic compounds [109,110]. Studies of halloysite indicate that it is a nanomaterial that can be considered safe and biocompatible. HNTs do not cause cytotoxic or inflammatory effects and can be safely mixed into medical materials such as dental preparations, bone cements and antibacterial coatings. Halloysite is also combined with dental resin composites (RDC), which are the most important coloured fillers for permanent teeth. HNT improves microhardness, maximum polymerisation rate, flexural strength, biological, and bioactive properties, as well as aesthetic considerations in such materials [105,109,111].

### 2.4. Composites

Composite materials are composed of at least two components with different properties, the appropriate combination of which allows the use of their best features and compensates for their drawbacks. Composites can be divided into the following groups: polymer matrix composites (PMC), ceramic matrix composites (CMC), metal matrix composites (MMC) and fibre-reinforced composites (FRC) [29].

Polymer matrix composites are materials resulting from the combination of several materials that differ in type and chemical composition while maintaining cohesion and uniformity. PMCs make it possible to reduce the weight of the finished product while maintaining its relevant properties, as well as improving resistance to corrosion, external active and passive forces. Polymeric matrices can be divided into two basic groups—thermoplastics and duroplastics. Examples of compounds belonging to the first group are styrene polymers, polycarbonate, polyamides, thermoplastic polyesters, and polypropylene. Duroplastics include chemically and thermosetting resins, among others.

Ceramic materials have several advantages such as high strength and high stiffness at very high temperatures, chemical inertness, and low density. With ceramic matrix composites, the disadvantages of ceramic materials, such as lack of toughness, failures in the presence of surface or internal flaws, high susceptibility to thermal shock, and the ease with which they are damaged during production and service, can be reduced at the same time as obtaining the advantages mentioned above. One of the main methods used for CMCs is to harden the ceramics by incorporating fibres, which are prioritised to carry the bulk of the load. The load distribution is dependent on the ratio of the elastic modulus of the fibres and the matrix, which is low for CMCs. Ceramic matrix composites have severalmechanisms for increasing their strength, such as compressive pre-stressing of the matrix, which causes axial compressive stress on the matrix after fabrication; crack impingement—crack arrest or bending due to higher resistance to cracking of the second phase (fibre or particle); fibre pullout—fibres with high resistance to transverse cracking cause damage along the fibre/matrix interface, leading to fibre pullout under further stress; crack deflection, where small fibre/matrix interfaces are able to deflect the propagating crack away from the main direction; and chase transformation toughening—the stress field of the crack tip in the matrix causes the fibres at the crack tip to undergo a phase transition, resulting in a volume increase that can squeeze out the crack. In CMCs, due to the limited toughness of the matrix and the high manufacturing temperature, thermal mismatch and compatibility problems between components are common problems, and have a very significant impact on the performance of these composites [112].

Metallic matrix composites comprise a continuous metal or alloy matrix and reinforcement. MMCs can be divided in terms of the reinforcements used into the following groups: particle-reinforced MMCs; short-fibre or whisker-reinforced MMCs; and continuous-fibre or sheet-reinforced MMCs. In determining the final properties of a metallic matrix composite, the phase boundary region is very important. Chemical, mechanical, thermal, and structural factors have an important influence on the nature of bonding of the composite components. MMCs exhibit a higher Young’s modulus compared to metallic materials. These composites are characterised by several strengthening mechanisms: strengthening of the Orowan; strengthening of the grain and substructure in the metal matrix according to the Hall–Petch-type relationship; strengthening of quenching with thermal deformation in the matrix; and hardening of the matrix by working. One of the main disadvantages of cast MMCs is post-porosity, which causes coring of the metallic matrix during solidification. In addition, with large fibre volume fractions, interdendritic fluid flow becomes difficult, and large-scale movement of the semi-solid metal is impossible. Discontinuities may also occur at the composite interface due to different types of bonding between composite components; differences in crystal structures and network parameter between matrix and reinforcement; differences in elastic moduli; differences in coefficient of thermal expansion; and thermodynamic imbalance between matrix and reinforcement at the interface [113]. 

Fibre-reinforced composites are capable of achieving high strength and stiffness. Their properties are related to the direction of the fibres. Unidirectional continuous FRCs exhibit anisotropy and achieve high strength in one direction, resulting in an advantage in specific applications through appropriate design [114]. The use of stranded, or bidirectional, fibres imparts orthotropic properties, while the use of chopped fibres (unoriented) imparts isotropic properties. The orientation of the fibres in FRC also affects the thermal behaviour of the material—depending on the direction of the fibres, the thermal coefficient changes. The properties of these composites also depend on the type of fibres, their relationship to the matrix and the quality of the fibre and resin impregnation [115]. 

In dentistry, polymer composites are considered to be a better substitute for metals, not only because of their lighter weight, but especially because of the easy corrosion of metals in the body and their possible toxic effects over a period of time, as well as causing allergies, ion release or aesthetic considerations [29]. To improve the mechanical properties of polymers, fibres and ceramics are added as reinforcements. With advancing science, nanocomposites are being used more and more (Table 2). These make it possible to improve the properties of individual materials and also to give them properties that enable the manufactured components to meet medical and dental requirements even better [116]. As dental materials, bionanocomposites mimic the properties and structure of natural tissue—they can withstand the oral environment with sudden changes in temperature or osmotic pressure and under high biting force. Polymeric matrices are used for the production of dental bionanocomposites, as they are a potential biological carrier for the recruitment of resident cells. Examples of such matrices include polylactide (PLA), polyglycolic acid (PGA), polycaprolactone (PCL), polylactide–polyglycolic acid copolymer (PLGA), polyether ether ketone (PEEK), or amphiphilic co-network(APCN) [117,118,119,120]. Inorganic nanomaterials are introduced into the composite to improve mechanical and surface properties, as well as to improve cellular response. The introduction of nanostructures into dental composites has significantly improved their properties, due to their having a larger surface area with the availability of more binding sites, and a particle size closer to that of the polymer molecules, so that intercalation with the polymer matrix can occur. Another advantage is that the particle size is smaller than the wavelength of visible light (400–800nm), so the material has greater translucency, which has a positive effect on the aesthetics of the detail. The restoration is also smoother and glossier. Compared to other types of resin composites or glass ionomer cements, they are characterised by higher compression, flexural and tensile strengths. In addition, they have the same abrasion resistance as natural human enamel. The most commonly used nanoparticles in dentistry are summarised in the table below [121,122].

Although biopolymers possess biocompatibility, they lack the necessary cellular characteristics. To overcome these limitations, bionanocomposites with inorganic nanomaterials have been produced. As a result, such composites show beneficial interactions with the cell membrane, spatially controlled protein binding for cell adhesion, nucleation of the mineralised matrix, or delivery of factors that promote stem cells developing into specific lineages. Inorganic nanoparticles have influenced the differentiation state of stem cells by acting as nucleation sites for the deposition of mineralised matrix [13,117]. They also play a key role in complementing and maintaining the mechanical properties of polymer matrices and promoting cellular activity. Bionanocomposites have been proven in clinical trials to be able to replace native tissues [126]. Despite their many advantages, it is also important to note the potential risks to the body caused by nanoparticles. They can cause oxidation by increasing reactive oxygen species (ROS) and also damage cell membranes through perforation. Other possible cytotoxic effects include damaging cytoskeletal elements, which disrupts cell division and intracellular transport, accelerating mutagenesis by inducing transcriptional disruption and DNA damage, and damaging and disrupting mitochondrial metabolism, thus upsetting the energy balance of the cell. In addition, nanoparticles may interfere with the formation of lysosomes, thereby impeding autophagy and macromolecule degradation and triggering apoptosis. Structural changes of membrane proteins are also possible, resulting in impaired transport of substances between the cell and the environment and intercellular transport. Nanofillers are also capable of activating the synthesis of inflammatory mediators, thus disrupting the normal metabolic mechanisms of cells, tissues and organs [123,125].

Hydrogel composites are also used in a wide variety of medical applications. Owing to their properties, they are perfect for use as drug carriers, as they exhibit important properties such as large specific surface area, safety for the organism, closed charge efficiency, biodegradability, and an extremely important ability to cross organismal barriers (e.g., PAMAM dendrimers are able to cross the blood–brain barrier) and cell membranes [129]. Their application enables prolonged drug release in targeted therapies. On the basis of intelligent polymers, systems have been developed that are able to release drugs based on various stimuli such as small changes in pH, light, temperature, and electric or magnetic field. Nanofibre-reinforced hybrid composites are of great interest in this field, as they are characterised by high surface-to-volume ratio, high drug encapsulation capacity, high drug loading, and stability. These features demonstrate the high potential of these materials as drug carriers [130,131,132]. Examples of applications include the delivery of hormones, e.g., in diabetes or cancer therapy. Interestingly, the interconnected porosity of the fibres not only ensures excellent cell binding, but also reduces the metastasis of cancer cells (locally controlled systems). Studies have shown that such gel nanocomposites reinforced by carbon nano-onions (CNOs) are capable of prolonged drug release without interfering with cell proliferation or inducing cytotoxicity, while being controllable by stimuli [133,134]. Other advanced drug delivery systems for cancer therapies take a similar approach. It has been noted that cancer tissues exhibit abnormal temperature gradients and are extremely sensitive to higher-temperature environments (40–43 °C), which is one of the reasons for the interest in thermoreactive polymers [135,136]. A promising approach to the issue of carriers is the incorporation of natural components such as bovine serum albumin (BSA), chitosan, gelatin, or silk carrier systems, among others. Protein nanofibres have particular potential, as they exhibit high surface area-to-volume ratio, high encapsulation capacity, stability, non-toxicity and non-antigenicity, high drug loading, and relative ease of scale-up during production. Focusing on albumin as a potential carrier, many advantages of this system can be noted. Through interactions such as electrostatic and covalent interactions and non-covalent coupling, a wide variety of drug molecules can be embedded in this protein. Furthermore, albumin exhibits adhesiveness, non-immunogenicity, high availability, cost-effectiveness, and biodegradability. Moreover, a composite of BSA with CNO and an attached anti-tumour drug in studies on mammalian cell cultures showed cytocompatibility, adequate release, and the prepared system was able to respond to dual stimuli [137]. Also of interest are hydrogel composites with a natural plant-derived protein, zein. This protein is hydrophobic and insoluble in water. Protein zein hydrogels containing poly 4-mercaptofenyl methacrylated CNO show high drug release over a wide range of pH values. It is also a sustained-release drug that, due to its properties, is able to release its cargo only in the intestines, which is a great advantage, as many substances are not resistant to the acidic effects of the gastric environment. This pH-dependent system also improves cell proliferation and does not induce cytotoxicity [138,139].

### 2.5. Manufacturing Techniques

There are many different methods for manufacturing and processing products for medical and dental applications. Notable among these are methods such as additive manufacturing and 3D printing. The production of dental components is becoming increasingly automated through the use of computer-aided design (CAD) and computer-aided manufacturing (CAM) systems. Initially, parts were manufactured using subtractive manufacturing (SM), which involved removing material from a block to create the desired shape. A much better approach is additive manufacturing (AM), which builds a part by adding material layer by layer based on a 3D computer model [7]. With this method, parts can be made from metals, ceramics, polymers, composites, and also from materials of biological origin. AM focuses on rapid prototyping and the rapid manufacture of end products in small to medium quantities. As an effective rapid prototyping technique, it allows highly individualised models to be obtained. For biomedical applications, it is possible to make many types of products with specific shapes and properties such as dental platforms, drug delivery systems, medical devices, dental and orthopaedic implants, tissue scaffolds or artificial organs. In the context of dental applications, AM is potentially able to enable the production of customised dental devices with grid-like shapes and intricate details. Furthermore, by omitting the design stage, the manufacture of complex components alone does not significantly increase production costs. The technology also reduces production time, as the dental device is directly manufactured using 3D scanning of the mouth [7,102]. An undoubted advantage of this method is also the reduction of human error in the procedures, as the production consists of far fewer steps compared to the methods used previously. Thanks to the fact that it is an additive technique, material waste and energy consumption are reduced, and the use of standard production tools such as cutters or drills is unnecessary. As a result, it can be said that AM enables the transition from mass production to mass customisation, with a reduction in production costs while increasing productivity. With this technique, dental components such as crowns, bridges, models, implants, scaffolds, dentures, surgical guides, or orthodontic materials can be produced. It is also possible to produce titanium implants with porous or rough surfaces [8,140]. Flexibility in terms of production is also among the advantages, as some AM machines are able to print multiple materials at the same time without having to change the structure during the manufacturing process. Despite the undoubted advantages, the technique also has some limitations. The incomplete reliability of the process, problems with the surface finish of the samples and the density of the materials must be noted. There is often a stepped effect on the manufactured products. AM technology is also underdeveloped for dental ceramics, mainly due to problems with the appropriate surface finish of the components, maintaining adequate mechanical properties and dimensional accuracy. Furthermore, due to the high melting points of ceramics, it is difficult to melt them using standard heating methods. On the other hand, cooling results may lead to thermal shock, as a result of which cracks may develop in the material [141,142].

Three-dimensional printing is a rapidly developing additive technology that is widely accepted in dentistry. Compared to conventional methods, 3D printing offers the advantages of process engineering. Thanks to the expiry of numerous patents, this method is now developing rapidly. It allows materials such as polymers, metals, ceramics, or composites to be manufactured using various techniques. The oldest and most commonly used 3D printing method in dentistry is VAT photopolymerisation (VPP) [143]. This method is based on the layered structure of a product made of a UV-sensitive liquid monomer, which solidifies and polymerises under the influence of a laser. Another commonly used technique is projection digital light processing (DLP). In this method, the device contains a microsystem with a rectangular array of mirrors, which is called a digital micro mirror device. Comparing this method with VPP, a definite advantage is that the execution time is not dependent on the number of objects or the geometry of individual layers, as each layer can be cured with a single shot of laser exposure, due to the patterned laser light produced, and not, as in the case of VPP, by scanning each area sequentially with a laser. The printing process in both methods can be divided into three discontinuous stages: exposure, platform movement, and resin replenishment [143,144]. However, since no actual printing takes place in the last two stages, a new method has been developed—continuous liquid interface production (CLIP). This allows continuous printing and speeds up the process. The monomers used in these methods should be of low to medium viscosity, as the use of more viscous monomers can result in poorer mechanical properties of the product. To counteract this, appropriate mixing methods should be used to obtain a homogeneous filler dispersion. VPP and DLP methods have limitations in processing several materials in one production process. The layer-by-layer technique also prevents products from achieving mechanical properties at the level of their monolithic counterparts. Although not ideal, these methods are the most advanced 3D printing technologies in dentistry [145]. Tooth models, customised implants, prosthetic teeth, transitional implants, bite splints, and orthodontic appliances are printed. Other techniques include photopolymer jetting and material jetting (MJ). In these jetting processes, the object is built up by a nozzle head with several in-line nozzles. In photopolymer jetting, a liquid photomonomer is used, which is then layered and cured with UV light. Material nozzles use a wax that solidifies thermally on a building platform. Because this process allows several print heads to be used simultaneously, it is possible to create products with different materials and colours that can exhibit a gradient of properties. When an item is manufactured using these methods, both the print resolution and the surface quality are very high. Another variant of photopolymer jetting is binder jetting. Thisconsists ofapplying a binder with the use of pressure nozzles on a powdered substrate. This method does not require additional support structures, but due to the complex geometry of products used in dentistry, its use in this area is very limited [9,143].Material Extrusion (MEX) is also a promising method. It works on the principle of thread extrusion—thermoplastic materials are supplied to the extruder as semi-finished products, where they are then melted and applied to the build plate through a die. A definite advantage of this method is its cost effectiveness and the fact that all materials that can be extruded can be used for production. It is also possible to produce objects with different material gradients by using several extruders [143,146,147,148,149,150,151,152,153].

The properties of manufactured parts largely depend on the material from they are made and chosen method [143,146,147,148,149,150,151,152,153]. However, the chosen manufacturing technique is also of great importance, as it also affects the characteristics and properties of the products, since even if the same material is used, it will have slightly different properties if different manufacturing techniques are applied. Therefore, it is very important to select appropriate manufacturing methods not only in relation to the materials used, but also with respect to the future characteristics and application of the manufactured elements. This phenomenon will be presentedon the basis of four techniques. The described properties are based on the manufacture of the same element—a ceramic scaffold for bone regeneration [154]. In the green machining technique, mouldings are produced by high-pressure powder pressing with a binder to provide raw mass strength and better machinability. In the green machining technique, mouldings are produced by high-pressure powder pressing with a binder to provide raw mass strength and better machinability [155]. In addition, it enables high ceramic solid loading to be achieved with uniform particle dispersion in the raw mass. In this process, the moulded part is machined first, followed by a sintering step [156]. The green machining method is very suitable for obtaining a better surface quality, as it can become much smoother and less rough after machining. The choice of solvent and the applied pressure also influence the products obtained using this technique. The application of this method makes it possible to obtain scaffolds with elastic properties consistent with those of bone, and to produce small part sizes as well as highly compacted scaffolds. The limitations of this technique are related to surface finish, dimensional accuracy and low freedom in pore design (only a spherical shape is possible) [154]. In addition, geometric boundaries are present, and the cutting tools used can cause textures and machining tears on the surface of the workpiece, which can initiate the propagation of microcracks [157,158]. Another method is binder jetting, in which components are formed by spraying alternating layers of powder and binder. The build material is aligned in the powder bed using a roller, and then the binder material is applied through a nozzle [154]. This technique allows the fabrication of dimensionally small cubic and cylindrical scaffolds with complex geometries. It also makes it possible to design pores with different geometric shapes and sizes and to obtain a highly rough surface finish without sharp edges [159,160]. There are a couple of challenges associated with this method. One of them is the difficulty of removing unbound powder material from small pores. In addition, some studies have confirmed the lack of dimensional precision, as well as the difficulty in loading them, which definitely reduced their potential in biomedical applications [160]. Another technique that provides different product properties is material extrusion (robocasting), in which a paste filament is extruded from a small ink jet that moves on a platform. A mixture of ceramic powder and binder is extruded to build up the part in the required shape layer by layer. This allows the creation of materials with controlled architectures and compositions [154]. The advantages of this method are the production of parts without the need for moulds or support materials. In relation to the material, this process enables good reproducibility in terms of both porosity and geometry adaptation to accommodate the gradual porosity of polygons and shaped walls. The process is also versatile and well controlled. The technique allows the creation of smaller pore and wall sizes [161,162]. Despite its undoubted advantages, the technique also has limitations. The lack of a support layer seems to strongly limit the parts produced in terms of geometrical freedom. The process also requires careful planning, taking into account many factors during fabrication, as small changes in these can cause cracks, binder removal, or excessive shrinkage [161,163]. The last technique presented in this comparison is laser-aided gelling (LAG). This is a powder bed technology in which a layer of a mixture of ceramic and binder is deposited on a surface platform and then irradiated with a laser or electron beam. This allows the suspension to be selectively scanned layer by layer to make the ceramic part. In this case, heat is induced by a laser beam, which initiates the chemical and physical gelation of the colloidal suspension. The solution then undergoes gelation and acquires stiffness, eventually forming a three-dimensional network, which is then sintered [154,164]. With this technique, it is possible to obtain scaffolds with a variety of geometries—cylindrical, cubic, prismatic, as well as geometries such as diamond and gyroid. Different pore shapes are also possible. The approach presented by this method allows the creation of a scaffold with interconnected porosities, which well mimics the natural architecture of spongy bone. Compared to the other mentioned techniques, this one makes it possible to obtain the required geometry with a specific degree of porosity at the same time [165,166,167]. Despite good results, LAG is still an underdeveloped method, for reasons such as the fact that the use of a laser beam at high melting temperatures leads to a high energy input. Furthermore, the CO_2_ fibre laser usually used for ceramic processing presents difficulties, as the waveform induced by it can be absorbed by most ceramics, making it unsuitable for laser sintering of ceramics due to the melting–solidification mechanism [154,162,168].

3D printing is a method that is constantly evolving. This technique, thanks to reaching a high level, is finding more and more applications in many branches of industry and human life. This method allows the printing of drugs in the form of tablets capable of disintegrating in the human body environment [169]. In addition, 3D printing now makes it possible to print complex structures while maintaining quality. It is possible to print smooth, rough, and porous structures, depending on the application requirements of the product. The technique allows simultaneous printing with different materials and the production of a component with a gradient of properties. Continuously improved computer processing systems allow structures to be generated for printing from 3D scans, which in the case of medical applications is a huge advance in personalised medicine. In this field, 3D printing allows the construction of spatial systems used in tissue engineering, forms of drug carriers in various forms, implants or films with a therapeutic substance capable of dispersing in the oral cavity [143,170]. Despite the significant achievements in this technique, there are still several challenges in developing these methods and raisingthe quality of products to an even higher level. One of the main challenges is to develop filaments with the right properties for specific applications. Quality features that can be improved in films include consistent dimensions, flexibility and stiffness, anduniform distribution of active substances (in the case of printed drug carriers. Printing of medically relevant components also faces challenges related to stability, quality, and safety issues when applied in the body. The porosity variations that occur in some techniques due to differences in adhesion between successive layers is also an issue that needs to be addressed. Challenges for additive techniques therefore include the control of design parameters, equipment performance, and the biocompatibility and sterilisationof the printed material. Furthermore, the fragility of the printed objects combined with the complex nature of the structures requires very well-planned structures [170]. 

Table 3 brings together examples of commercial products used in 3D printing techniques.

The current standards distinguish seven processes. These are Vat Photopolymerisation (VP) (a process of layered photopolymerisation until a defined volume is obtained, using a concentrated UV light beam); Material Jetting (MJ) (a technology of layered printing of liquid material on a model, which uses layered sections. The liquid–solid phase change usually takes place through solidification or photopolymerisation); Binder Jetting (BJ) (the process involves bonding a powdered material with a liquid binder. The material is bonded by means of a liquid binder deposited from the print head onto a cross-section of a layered model); Powder Bed Fusion (PBF) (a process involving the joining of powdered material in a selective manner. This is done by using thermal energy to selectively melt the layers of the powder bed); Material Extrusion (MEX) (a method of extruding layers of material—a thermoplastic material is extruded into a fibre, which is layered according to digitally determined paths); Directed Energy Deposition (DED) (a process consisting of the targeted melting of the supplied material. In this method, concentrated energy (laser beam, electron beam or plasma arc) melts the material in layers as it is deposited); Sheet Lamination (SL) (this is lamination in cross-section. In this process, successive sections of the model are cut out of sheets of material and then glued together). Despite the multitude of techniques, not all of them are directly applicable in medicine; however, it is possible to use them in an indirect approach.

Although additive manufacturing and 3D printing are the leading techniques for component manufacturing in modern medicine, there are also other methods for manufacturing biomedical devices. One example of such a technique is casting. It is a technique introduced to better fit biomedical devices to the patient compared to mass-produced ones [172]. However, it is not widely used at present for several reasons—the fit is better than with mass production, but still lower than with computer-aided machining systems. Furthermore, it is a time-consuming method, requiring a lot of experience and a multi-step quality control process [173]. It is a technique that is error-prone at many stages, and often the manufactured components are distorted or simply do not fit the patient very well. For these reasons, the use of this method for biomedical purposes is being abandoned, primarily in favour of additive manufacturing methods [172].

Another technique is foaming, which enables the creation of porous structures. Gas foaming is an efficient and inexpensive technique that enables the creation of porous 3D structures facilitating tissue regeneration [174,175]. The principle of gas foaming is that the supercritical fluid acts as a plasticiser and lowers the glass transition temperature and/or melting point, thus creating porosity within the 3D structure [176]. The released carbon dioxide interacts with many polymers, leading to their plasticisation even at moderate or low pressures [177]. This method is only suitable for amorphous or semi-crystalline polymers, as it is not able to lower the glass transition temperature of crystalline polymers [175,178]. Unfortunately, there are also limitations associated with this method. The lack of interconnection of the resulting pores means that cell ingrowth is limited. Furthermore, this method also does not allow close control of the resulting structure [179].

Another method for producing biomedical components is hot stamping. Hot stamping involves the simultaneous application of heat and pressure to a mixture of powders [180]. This technique leads to metal components with high mechanical parameters and small and homogeneous grain size, but which are characterised by low porosity. The advantage of this method is definitely the possibility to create composite elements such as metal–ceramic, avoiding both the decomposition of ceramic materials and the subsequent separation of ceramic coatings from the metal surface. This method allows good distribution of bioactive sites (ceramics) in the metal matrix, as well as good consolidation of the matrix [181]. This fabrication method, despite its advantages, is also not without disadvantages. Currently, it is being replaced by newer additive manufacturing methods, such as selective laser melting, which obtains elements with better quality [182].

## 3. 3D-Printing-Based Skull Reconstruction Surgery: Challenges and Material Solutions

Regarding the use of 3D-printing technology for medical purposes, one of the most demanding areas of application is the reconstruction of the bony structures of the skull. The preparation of high-fidelity human skull imitations for demonstration/educational purposes is already difficult enough, due to the extremely complex and subtle composition of the individual bones as the jigsaw pieces of which the whole skull is composed [183,184,185,186,187,188]. The level of difficulty increases manifold if such a replica is going to be implanted as the surrogate for the patient’s own bone during asurgical procedure. The need for skull bone substitution may occur on two main occasions. First, skull reconstruction (also described as cranioplasty, from the ancient Greek krānìon = ‘skull’ and plastòs = ‘moulded, formed’) may become part of primary operations during which part of the skull must be resected due to its being affected by pathology of various characters (e.g., neoplastic tumour, bone infection not responding to antibiotic treatment, or bone dysplasia causing abnormal growth) [189,190,191,192,193,194]. Second, the bone-replacing material may be implanted during a follow-up, i.e., secondary, procedure if for any reason part of the skull needed to be removed temporarily and its reconstruction during the same surgery was not feasible or was undesirable. The flagship situation here is cranioplasty secondary to decompressive craniectomy (DC). DC is the urgent neurosurgical intervention during which a life-threatening increase in intracranial pressure (ICP) (caused by, e.g., therapy-resistant brain oedema resulting from trauma or ischemic stroke, intracranial bleeding, infection or decompensation in course of progressive intracranial tumours) is relieved by removal of a large part of the calvaria (i.e.,the part of the skull directly covering the brain hemispheres) [195,196,197,198,199,200,201,202]. This procedure is extremely effective at lowering the physical parameters of ICP [197,203,204,205]; however, it also carries the risk of several drawbacks, since the brain afterwards is covered only by soft tissue layers. Thus, the cerebral hemisphere, lacking the rigid protection of the skull, is exposed to external pressure and its changes, as well as being jeopardised by mechanical damage when even minor knocks, bumps or blows to the area denuded of bone occur [206,207,208,209,210]. Moreover, the lax brain tissue, if not encased in the closed box created by the skull, may undergo relevant deformations during postural changes, not only affecting its shape, but also resulting in brain dysfunction [211,212]. For the set of symptoms related to partial lack of the skull covering, the term “syndrome of the trephine” was coined [213,214,215]. Regarding this entity, the variety of symptoms encompasses some minor signs, such as mood changes, emotionallability, and memory and concentration deficits, and ranges up to focal neurological deficits including motor or speech impairment (aphasia) or even reduction of consciousness (including a comatose state in more severe cases) [216,217,218,219,220,221,222]. In light of this description, it is clear that the procedure of re-covering the previously decompressed brain by means of cranioplasty is not mere cosmetic surgery, but rather a crucial and dramatic step of restoring the biophysical and mechanical properties of the human skull with the aim of optimising the intracranial environment for the sake of proper function of the brain tissue [223,224]. For this reason, the choice of the material intended to replace the lost bone fragment is a difficult task, as it needs to satisfy several criteria specific to the skull, as the anatomic and biophysical unit, intended to carry the implant. 

The first difficulty is related to the anatomical properties of the skull bones. The human skull consists of 22 bones (8 bones composing neurocranium—the part of the skull covering brain—and 14 bones comprising the viscerocranium, i.e.,the facial skeleton). Each of these bones demonstrates a shape that is unique, both in regard to other parts of skeleton (in contrast to, e.g.,the repetitive shape patterns of the long or short bones of the extremities) and in regard to differences between individuals. More so, the irregular skull bones contain numerous indentations, pneumatic cavities, and orifices (foramina) through which the crucial anatomical structures, including cranial nerves, blood vessels, and parts of the central nervous system (CNS),pass. Clearly, these irregularities need to be closely duplicated in the process of pre-paring the bone implant. In cases where such an implant needs to contain cranial foramina, a two- or multi-piece technique may be required. Second, the mechanical properties of skull bones need to be mentioned. Even if the shape of the calvaria (composed of flat, curved bones) is less complex than in the case of shape irregularities seen in case of facial bones, this part of the human skull possesses a specific composition, enabling it to resist mechanical forces including loading, shearing, and indentation forces, and thus fulfil the main task of protecting the CNS and the sense organs [225,226]. The specific mechanical properties of cranial flat bone architecture (simplifying, composed of a cancellous bone layer called diploë, encased between the inner and outer layer of solid cortical bone) are difficult to mimic using (usually homogeneous) compounds [227,228,229]. Finally, while considering the appropriate material for skull bone substitution, the relatively large surface of interaction between the surrounding tissue and the implant needs to be considered [230]. Thus, the potential toxicity of material and the probability of local or systemic foreign body reactions is distinctly higher than in bone-replacement techniques outside the neurosurgical or craniofacial surgery domain [231]. To date, besides metal bone implants (usually made of titanium), several ceramic and polymer materials fulfil the strict criteria of mechanical durability, possibility of free pre-forming (including the option of creating elaborate, three-dimensional replicas of irregular bony structures), and high biocompatibility. Among them, polymethylmethacrylate (PMMA), polyetheretherketone(PEEK), polypropylene–polyester knit (PPK), and hydroxyapatite (HA) have been found to be suitable for producing the individualised, computer-aideddesigned (CAD) models of skull parts to be used for surgical use during cranioplasty surgeries. 

### 3.1. Polymethylmethacrylate (PMMA)

Of several polymer materials, PMMA, a mouldable acrylic resin, has the longest history of use as a bone equivalent material, including in cranial surgeries [190,232,233]. This material is characterised by mechanical resistance similar to (or higher than) the original bone, offering sufficient biophysical protection to the brain [234,235,236]. The medical/biological advantages of PMMA include high biotolerability due to low chemical reactivity [237,238]. Due to the lack of porosity (if used in its typical form), PMMA is considered to be biologically inert and not infiltrated by scar or bone by means of tissue scaffolding [232,239]. Surgical PMMA is available as a two-compound bone cement polymer, where after mixing the fluid and solid components, an exothermic chemical reactionis initiated, during which PMMA changes its properties, transforming from being an easy-to-form, plastic, semi-fluid material to a solid form [240]. For this reason, the career of PMMA as a cranioplastic compound began with the preparation of hand-made skull implants in situ or during the progress of surgery. Certainly, this technique is burdened by the limited anatomical fidelity of the replicas, as the proper shape of the implant strongly depends not only from the dexterity and experience of the surgeon, but also on their level of “sculptor” skills [241,242,243]. For this reason, PMMA was quickly identified as a material apt to serve for the preparation of implants with the use of 3D-printing techniques. Importantly, the advantage of the availability of PMMA as a sterile bone cement has impelled the development of 3D-pre-printing techniques, in which not the implant itself, but a surgical mould to be used as a template for the intraoperative casting of the implant is 3Dprinted. With the use of this 3D-molding technique, multiple uses of the same cast during the same procedure, e.g., in cases where the primary replica gets damaged during the final fitting to the operative site, is possible [244,245,246,247,248,249]. Moreover, if the mould is made of material that may be re-sterilised, it may be reused in the rare cases of follow-up or revision surgeries. 

Certainly, PMMA, if used for 3D-based cranioplasty, is not completely void of side effects. Despite its biological and chemical inertness, the tolerability of this material is somehow reduced by the risk of implant infection [250,251,252], aseptic reaction to the compound [253], and allergic reaction to PMMA [254]. Additionally, cases of mechanical failure, including breach of the implant due to direct injury or fall, have been documented [255].

### 3.2. Polyetheretherketone (PEEK)

A more modern polymer compound, PEEK, was adopted for biomedical use, including cranioplasty techniques, in the 2000s [239,256]. In contrast to PMMA, its implementation is reserved rather to the field of pre-fabricated implants; however, due to its high bioadaptability, including the ability to produce porous implants able to serve as the scaffold for subsequent scar and bone growth, and its excellent physical properties (including mechanical robustness and appropriate thermal conductivity [77,257]), it has attracted continuous interest from craniofacial surgeons and neurosurgeons for performing cranioplasty procedures [232,233,239,256]. One of its main advantages is its ability to be used for manufacturing bone replicas of high spatial fidelity [258]. This property is particularly important in cases where the implant needs to be pre-printed on the basis of the surgical resection plan [259]. In general, PEEK is well tolerated by surrounding tissue; however, cases of aseptic fluid accumulation (seroma) and post-surgical infections have been documented in several studies [260,261,262]. On the other hand, its fair biocompatibility is confirmed by the successful use of PEEK cranioplasty even in cases with reduced implant acceptability due to post-radiation changes in soft tissue [263]. In general, the manufacture of ready-to-use PEEK skull implants using 3D-printing techniques has become widely available, and one of the major drawbacks (the need for in-house sterilisation) has been overcome by the production techniques employed by most commercial biomedical suppliers. 

### 3.3. Polypropylene–Polyester Knit (PPK)

One of the materials with high biological compatibility, but which is less commonly used as cranioplasty compound, is PPK. The use of PPK implants in vascular surgery (as the vessel prosthesis [264]) or in abdominal surgery (PPK mesh for hernia repair [265]) has been introduced previously, and due to high mechanical robustness of the material, PKK has been accepted in the production of cranial implants (initially produced as standardised pre-shaped implants of different size and curvature, intended for intraoperative tailoring) [266]. In the 2010s, a 3D-printing technique was adapted in the production of PPK implants, and the successful use of individualised CAD-based skull implants has been documented in dozens of cases [267,268], including in large cranial defects [269,270]. PPK in its cranioplasty-adapted version seem to remain a chemically inert, yet biologically active compound, which becomes incorporated in the recipient’s tissue by means of modest foreign body scarring [271,272]. One of the factors defining its growing popularity is the convenient economic profile of the material itself, as well as its production process [273], making PPK implants most affordable for even less prosperous health care and insurance systems. 

### 3.4. Hydroxyapatite (HA)

When comparing different polymer materials with respect to their chemical similarity to natural bone, the porous mineral material HA demonstrates the most accurate structure and composition. Micro- and macroporous HA is characterised by its high biological compatibility with the bone, including its involvement into cell-mediated processes through the incorporation of the newly implanted bone replica [274]. The proliferation of microvessels and osteomodelling activities (including osteointegration due to the modelling activity of osteoclasts) [275] has been declared to be proof of the superiority of HA as a substrate for the production of implantable skull replicas [276,277,278]. Additionally, this mineral material is suitable for 3D-print-based manufacturing [276,279,280]. Several drawbacks are related to the mechanical properties of HA-made implants. First, the ability to further adapt the pre-fabricated implant for the surgical site is somehow reduced by its brittleness [281]. This same disadvantage is also relevant with respect to the risk of implant fracture during the carrier’s activities after surgery [282,283,284,285,286]. This drawback seems to be compensated by the reported ability of HA fracture lines to heal due to its high biological activity and due to its similarity with the mineral composition of human bone [278,287,288]. 

### 3.5. General Considerations

Certainly, the list of synthetic materials potentially serving as compounds for the manufacture of implantable skull replicas is not closed. Several attempts of the experimental use of polycaprolactone-β-tricalcium phosphate (PCL-TCP) [289], multimaterial hydrogel scaffolds [290], or copper-doped bioactive glass scaffolds [291] have been performed successfully in recent years, yielding promising results with respect to the adaptation of these materials for clinical use. However, regardless of developments in biomaterial engineering, one important pre-requisite remains crucial for further progress in the area of cranioplasty materials: new compounds should be suitable for the manufacturing of 3D-printed implants. The use of 3D-printing techniques has become an absolute must for the optimal planning and efficient conduction of cranioplasty procedures. Using pre-fabricated, individualised implants definitely shortens the time required for the procedure and increases the anatomical fitting of the bone replica to the defects, thus generally raising the chance of the therapeutic success of the procedure and reducing the risk of revision surgery [239]. Moreover, the trend of establishing point-of-care availability of 3D medical printers [244,258,292,293] could possibly shortens the time between obtaining the computer tomography scans, usually applied as a template, and receiving a ready-to-use CAD-based implant(or implant mould). In summary, the development of 3D printing procedures has significantly facilitated pre-paring and conducting cranioplasty treatment. The new materials should be characterised not only by high biocompatibility, mechanical robustness similar to bones, and the possibility for integration into the living surrounding tissue, but should also suit the requirements of 3D printing technology in order to allow the highest level of anatomic adaptation to the needs of individual patients (and surgeons).

## 4. Nanocomposite Transitional Implants for Guided Bone Regeneration

This section presents the use of the materials discussed in the first section, primarily composite materials and nanocomposites, for dental implants, focusing in particular on transitional implants and their properties.

A dental implant is a small screw placed in the jawbone that replaces an extracted or lost tooth root. A tooth crown is mounted on it, the shade of which may be adjusted to match natural teeth. It performs not only an aesthetic function, but also carries the load of eating.

Transitional implants are also called scaffolds and are used for bone augmentation, i.e., guided bone regeneration. The reason for their use is the lack of adequate height and/or width of the alveolar bone, which prevents the application of a permanent implant. The aim of augmentation is to promote bone growth while limiting the growth of rapidly growing soft tissues by appropriately loading the alveolar process and providing a scaffold for the emerging bone. By using biodegradable and bioresorbable materials, the need to remove transitional implants is also avoided, which is a definite advantage [294,295].

**Figure 6:**
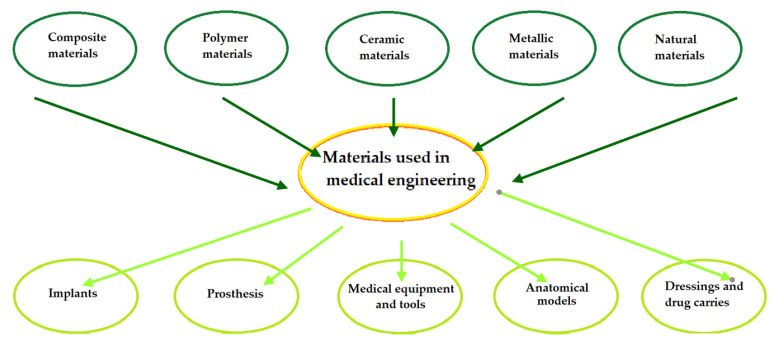
Material classification scheme.

Dental transitional implants must meet several requirements in order to perform their function well. The most important of these is the use of materials with good mechanical properties. Such biomaterials should also be characterised by high biocompatibility, bioresorbability, controlled rate of biodegradation, bioactivity, and mechanical strength at a sufficiently high level. Biocompatibility is mainly responsible for non-toxicity to host tissue and not causing allergic reactions or inflammation. Bioactivity should support normal cellular activity and promote osteoconductive and osteogenic activity as well as angiogenesis. For mechanical properties, it is important that the transitional implant be able to withstand mechanical loading and stress and possesses similar mechanical properties to natural oral components. Biodegradability and bioresorbability make it possible to control the rate of resorption of the scaffold material into the newly formed bone tissue. They also allow variations in the rate of material degradation and controlled rates of drug delivery and bioparticle incorporation. Controlling the rate of biodegradation can be done by influencing scaffold-dependent parameters such as the manufacturing methodology, its size and shape, the roughness of its surface, surface-to-volume ratio, surface porosity and pore size, and additives or the presence of impurities (Figure 6). 

**Figure 7:**
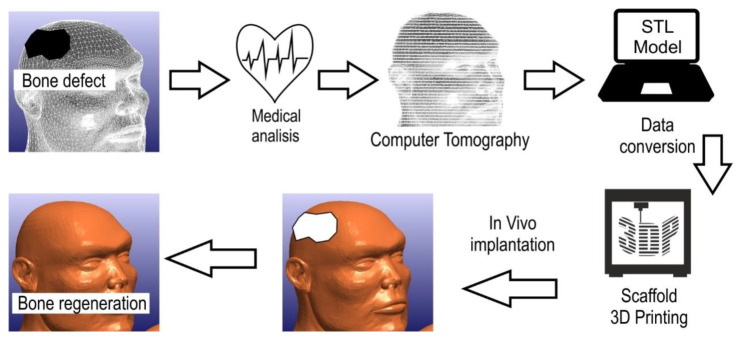
Scheme showing the procedure for manufacturing scaffolds for guided bone regeneration based on [300].

Other factors are related to the environment in which the transitional implant will be placed—in this category, the pH and ionic strength of the environment, temperature, mechanical loading, tissue remodelling, enzyme concentrations, and the site of implantation all have an impact on degradation. Another requirement for transitional implants is the appropriate design of the engineered scaffolds, as different types of structures and architectures are used in bone tissue engineering. Design has a significant impact on the mechanical properties of the component. Porosity, as well as pore size and shape, is an important factor for cell attachment and rapid growth. Transitional implants can meet these requirements because the three-dimensional porous scaffolds provide a large surface area for bone growth as well as high transport rates of both nutrients and waste. For this reason, a porosity of approximately 90% is recommended for transitional implants. To allow adequate diffusion of nutrients and cell survival and proliferation, the minimum pore size is 100 μm. However, pore sizes in the range of 250–350 μm are required to allow bone tissue ingrowth. Although high porosity can have negative effects on the mechanical properties of the scaffold, multi-scale porosity with a combination of micro- and macropores is optimal for the scaffold to perform its function. The appropriate rate and behaviour of the material during degradation is also an important requirement, and attention should be paid to this when choosing nanofillers, as they can affect the speed at which this process occurs. Another important factor is the adhesion between the substrate and the coatings, which can be defined as the adhesive strength [35,296,297,298,299]. Figure 7 shows a schematic of the manufacture and operation of scaffolds for guided bone regeneration:

Among the many bionanocomposites used in dental implantology, polymer–hydroxyapatite composites play a huge role, as the use of biodegradable polymers as matrix and HA as nanofiller offers excellent biomedical applications ranging from wound healing and tissue reconstruction to drug delivery. Table 4 lists the most commonly used polymer–hydroxyapatite composites along with manufacturing techniques [50,296].

The development of tissue engineering and its clinical application, i.e., regenerative medicine, requires the creation of pre-clinical models that can later be translated into clinical products. Such studies have been conducted to evaluate the concept of bone regeneration on animal models. Rodent models have proved successful in mimicking bone tumours, and models of larger animals such as goats, sheep, and pigs have been used to investigate bone formation and scaffolding effectiveness on induced tibial and femoral bone defects, providing significant clinical plausibility to human cases [338,339]. Translation of the results to the human body is possible, as the bone composition of animals such as dogs, goats, sheep and pigs have a similar bone composition to humans [340]. The most commonly used animals in studies of bone defect regeneration are sheep, because of the good documentation of mechanical loading of the limbs during walking, the similar body weight of adult animals to adult humans, and the similar rate of metabolic bone remodelling [339,341,342]. Research has shown that porous scaffolds grafted with bone marrow-derived stem cells (BMSCs) allow an increased rate of bone formation compared to ungrafted scaffolds [343]. Furthermore, in vitro studies have indicated that the osteogenic potential depends on the origin of the bone and varies for different bones [344,345]. Tissue engineering techniques used in vivo are primarily designed to study the properties of biomaterials including the ability to osteoconduction. Such models can be used to study the spread of metastases or bone colonisation. This allows us to understand the importance and level of interaction between bone and tumour cells [338,346]. The rabbit is also a suitable animal model. Study has shown that the use of PMMA membranes for guided bone regeneration of cranial defects provides great efficacy. They promote osteogenesis and increase biological activity while inhibiting soft tissue [347,348]. The use of intermediate pre-clinical models such as animal models and in vitro cultures is essential in research on scaffolds for guided bone regeneration. They allow the properties of scaffolds and materials to be studied, and their effectiveness to be tested. Such models also allow the understanding of the processes involved and provide a good representation of the processes taking place in human organisms. They also allow several conclusions to be drawn and devices to be improved as much as possible prior to clinical application in patients [338,349].

Developments in science and technology have made guided bone regeneration possible. In the case of dentistry, nanocomposite transition implants (scaffolds) are being developed which, thanks to their suitable properties, allow the growth of bone tissue while blocking the growth of soft tissue. The formation of bone allows the placement of a permanent implant without the risks associated with inadequate fixation in the dental pit. To limit the interference and possible undesirable effects of transitional implant removal, such as the risk of infection or tissue damage, scaffolds are made of biodegradable and bioresorbable materials [303,350]. Due to their excellent properties for these purposes, some of the most commonly used nanocomposite systems in oral bone reconstruction are polymer–ceramic nanocomposites, among which PLA/HA is the most common. Nanocomposite systems with HA in the form of nanotubes are able to mimic the natural morphology of bone apatite, since hydroxyapatite is identical to bone tissue—HA is the main mineral component of the bone matrix and also provides minerals for bone cells, enabling regenerative action. In addition, the addition of this mineral accelerates the degradation of the polymer matrix, as it has the ability to counteract lactic acid, which is secreted by the degradation of PLA and whose presence reduces the rate of degradation of polylactide. This improves the osteoconductive properties of the material and has a positive effect on the augmentation process [307,351]. Transitional implant scaffolds made of biodegradable PLA and HA are characterised by biocompatibility, favourable mechanical strength, relative ease of fabrication and good osteoconductive properties towards osteoblasts and mesenchymal stem cells—a very important factor from the tissue engineering point of view. The composite transitional implant exhibits better properties when the filler particles are on the nanometric scale. Nanohydroxyapatite not only exhibits higher bioactivity compared to its micro- or macro-particles, but scaffolds made of PLA/HA nanocomposite are characterised by higher interactions between the material and the cells, resulting in increased cell adhesion and better cell growth and differentiation [317,350,352,353]. Furthermore, hydrophilicity and water absorption are increased. An undoubted advantage of this system is that it can also be used in rapid prototyping techniques. Thanks to the combination of PLA/HA, it is possible to obtain nanofibrous three-dimensional scaffolds with a large pore volume and interconnections between them, which are an effective growth environment for pre-osteoblasts. An appropriate amount of nanofiller makes it possible to produce scaffolds with porosity exceeding 85%. An increase in HA content in the material improves hydrophilicity, mechanical stability and cytotoxicity; however, a decrease in porosity has also been observed [351,352,354].

## 5. Conclusions and Future Prospects

This review summarises the current knowledge of materials used in medicine and dentistry from the perspective of their use in the manufacture of transitional implants for guided bone regeneration. The development of modern medicine is not possible without the multidirectional use of natural and synthetic macromolecular compounds. Nanocomposite systems, in particular PLA-HA, currently seem to be the most suitable for these purposes. Thanks to their characteristics, they allow for significantly better results and areas of action hitherto unavailable for implants, as they were standardly made of metal. They allow the propagation of bone tissue growth, while restricting rapidly growing soft tissues. The appropriate structure also allows the gradual release of drugs, thus reducing the risk of infection or inflammation. After the time required for adequate bone growth, nanocomposites are capable of complete biodegradation. This is one of their most important features compared to implants used in the past, as metal implants showing osteoconduction remained in the body or required invasive methods in case of removal, some of them showing toxicity and other undesirable effects. The biocompatible PLA-HA system, during biodegradation and bioresorption, supplements the growing bone tissue, in addition to having an absence of toxic effects. Furthermore, good osteoconductive and osteointegrative properties have been confirmed for this system. Composite products are also not as susceptible to stimuli as components made of metal, among other effects caused by temperature factors. Important factors also include the lighter weight of composites and the possibility of a better fit to the respective site, both structurally and, in cases such as transitional dental implants, colour-wise.

The possibility of printing composite parts using additive manufacturing and, in particular, 3D printing, which is still being developed, also offers great prospects. These techniques make it possible to print from 3D scans. This is a huge advance in medicine as it allows the use of personalised components. Techniques used previously were not capable of producing personalised parts on a large scale with the level of precision required. Computer-aided 3D printing not only produces a unique, personalised part, but also reduces human error. Moreover, these methods make it possible to produce structurally complex elements, which thanks to their structure will have a much better effect on the body. 3D printers are also increasingly widely available, so the possibility of printing the necessary elements directly in hospitals or dental surgeries would definitely reduce the price and waiting time for scaffolding, while increasing availability. Manufacturing with these techniques also allows the creation of models, which definitely helps, not only in the training of doctors, but also with achieving a better understanding of the problem and the selection of appropriate treatments or operations in medical practice. This is because currently printed bone models offer a greater opportunity to understand the pathological situation in a patient than radiological diagnosis.

Despite the enormous number of advantages, these materials and techniques are still not perfect. They are currently being improved in order to produce elements with properties identical to their natural counterparts. This would not only allow perfect harmony of interaction with natural elements, or give ideal models, but would even allow damaged bones to be replaced without negative effects caused by the difference in properties. In this case, it is necessary both to find materials that meet all the requirements and to be able to use them in 3D printing. Additive manufacturing techniques are currently at a very good level, but there is still a need to improve these methods in order to obtain better quality structures and surfaces, especially in the context of medical applications. Improving computer processing programs is also a challenge. Research is being carried out that would allow missing elements to be rendered if they cannot be directly referenced, for example, on the basis of structure, alignment and feature points of the elements to be referenced. The authors of this manuscript plan to conduct future research into 3D printable biomaterials to produce scaffolds for guided bone regeneration, which will lead to advances in materials science as well as medicine.

## Figures and Tables

**Table 1:** Polymers for medical and dental applications.

Polymer	Purpose of Use	Biodegradation/ Bioresorbability	Biocompatibility	References
Poly-lactic acid (PLA)	Barrier membranes, drug delivery, guided tissue regeneration (in dental applications), orthopaedic applications, stents, staples, sutures, tissue engineering	+	+	[2,34,35]
Poly-glycolic acid (PGA)	Barrier membranes, drug delivery, guided tissue regeneration (in dental applications), orthopaedic applications, stents, staples, sutures, tissue engineering	+	+	[34,35]
Poly-caprolactone (PCL)	Long-term drug delivery, orthopaedic applications, staples, stents	+	+	[2,34,35]
poly(1,8 octanediol-co-citrate) (POC)	Mimics the mechanical properties of vessels, support the proliferation of human aortic endothelial cells while inhibiting the proliferation of human aortic smooth muscle cells in vitro, serve as therapeutic coatings to improve the long-term patency of transplants	+	+	[34]
Poly-lactic-co-glycolic acid (PLGA)	Barrier membranes, drug delivery, guided tissue regeneration (in dental applications), orthopaedic applications, stents, staples, sutures, tissue engineering	+	+	[34]
Polydimethylsiloxane (PMDS)	Uses for production of contact lenses	+	+	[36]
Polymethylmethacrylate (PMMA)	Masses for orthopaedics, surgery and dental prostheses, use in the production of intraocular lenses, gentamicin carrier in the treatment of infected joints	+	+	[1,2,36]
Polyethylene (PE)	Acts as a binder in prosthetics as an element of implants	−	+	[36]
Polyhydroxyalkanoates (PHAs)	Production of sutures, wound dressings, cardiovascular patches, orthopaedic pins, adhesive barriers, stents, guided tissue repair and regeneration devices, articular cartilage repair devices, nerve guides, tendon repair devices, bone marrow scaffolds	+	n/a	[2]
poly-β-hydroxybutyrate (PHB)	Long-term drug delivery, orthopaedic applications, stapes stents	+	n/a	[35]
Poly-para-dioxanone (PPD)	Used in the field of medicine in the form of films, foams, laminates, adhesives and surface coating	+	+	[35]
Polyhydroxyvalerate (PHV)	Long-term drug delivery, orthopaedic applications, stapes stents	+	n/a	[35]
Polyether ether ketone (PEEK)	In the area of implantation, it is used for artificial skull plates, elements of finger and knee joints and spine implants, more and more often in dentistry as an element of abutments, fixed prosthetic skeletons and skeletons of partial skeletal dentures, including precise fixing	+	+	[10,37]
Polyphosphazenes (PPZs)	Blood contacting devices, drug delivery, skeletal reconstruction	n/a	n/a	[35]
Polydioxanone (PDS)	Fracture fixation in non-load-bearing bones, sutures, wound clip	+	n/a	[35]
Polycarbonate (PC)	Blood separators, surgical masks, high pressure syringes, disposable dental devices used in artificial kidney dialysis	−	+	[2]
Polyethylene glycol (PEG)	Component of hydrogel dressings successfully used in the treatment of open wounds, they are tested in terms of treatment of nervous system injuries of increasing the effectiveness of gene therapy	+	+	[2]
Polyurethane (PUR)	Artificial organs are created, the creation of breast prostheses, an artificial heart, transplants, membranes, catheters, artificial skin, oesophageal prosthesis, channels for nerve regeneration, they began to be used in heart valves and ventricles and in aortic transplants	+	+	[2,36]
Polypyrrole (PPy)	A potentially electrically addressable tissue or cell support medium, neuroprosthetics, biosensors and drug delivery	+	n/a	[2,34]

**Table 2:** Nanoparticles used in nanocomposites for dental application.

Nanoparticle	Purpose of Use	Advantages	Toxicity	References
Carbon nanotubes	Coating of the teeth surface, teeth filling	Large surface area, adheres easily to the tooth surfaces and to the surfaces of dentin and cementum, bring active agents to live cells	Blocks potassium channels, accumulation in the hippocampus which induces oxidative stress, increased ROS factors, increased apoptosis factor	[29,117,123]
Graphene	Teeth coating, biofilm reduction, suitable for implantation	Treat bacterial biofilm, cost effectiveness, low dentistry form a uniform crystal lattice, fracture resistant	Toxicity depends on purity, shape, size and oxidative state,	[29,117,124]
Hydroxyapatite	Reduce dental hypersensitivity, retard auxiliary demineralisation, act as cavity filler, repairment of enamel surface, promotion osteoconduction	HA particles can easily integrate into the dental tubules, similar composition with teeth and bone, adsorbed to the enamel of the teeth, biocompatible, protect the teeth by making a film of artificial enamel around the tooth, reform periodontal shortcomings	Particles travelled to and dispersed into lungs, spleen and liver by blood, the inflammatory response, signalling pathway, induce oxidative stress,	[29,124,125]
Halloysite	Drug delivery, promotion attachment and proliferation of human dental stem cells, tissue engineering, scaffolds	Significant adsorption and loading capacities, improving mechanical properties, antibacterial, biocompatible, haemocompatible, sustained release of therapeutic agents, increased water adsorption and degradation rate	No toxic effects in recent studies	[126,127]
Zirconia	Reduces bacterial adhesion to the tooth surface, effective polishing agent, provide protection against dental carries	Similar mechanical properties and colour to those of a tooth, sensible biocompatibility, have low cytotoxicity, high fracture resistance	Significant DNA damage in human T-cells, induce apoptosis, inhibition of cell proliferation, nanoparticles can stop the cell cycle	[29,128]
Silica	Tooth polishing, an antibacterial agent, dental filling agent, prevents dental caries, to teat dental hypersensitivity	Biocompatible, low density, significant adsorption ability, low toxic effect, effective cost, reduces roughness of teeth surface (polishing agent)	Ability to induce silicosis, cytotoxicity, genotoxicity, possibility to induce oxidative stress, mediate apoptosis, g	[29]
Titania	Dental implants	Long-term effect on dental implants, surface modification—less bacterial adhesion, improved hardness, enhance the bone grow, protein adsorption and cell adhesion	Increased ROS factors, increased oxidative stress, genotoxicity, induce cellular apoptosis, increased inflammatory responses	[13,29,123,124,128]
Silver	Antimicrobial agent, dental implants, dental prosthetics, dental restorative material	Decrease bacterial colonisation, increases oral health, long-term antibacterial activity	Reduces mitochondrial viability, increased LDH release, increased ROS, up-regulated Bax protein expression, changes in astrocyte morphology, acute calcium response, induce apoptosis of lung cellular, increased cardiocyte deformity and lipid peroxidation, decreased levels of GSH, SOD and CAT, increased oxidative stress, increased release of inflammatory mediators in liver, inhibits mitochondrial ATP-ase in spleen	[13,29,117,123,124,128]

**Table 3:** Examples of commercial polymer products in the 3D printing process (Adapted with permission from Ref. [171]).

3D Printing Technique	Type of Material	Name of Commercial Polymer	Area of Application
MEX Fused Deposition Modelling (FDM)	Polylactide (PLA)	Resorb x	Used to receive surgical sutures and dental implants
	Polycarbonate (PC)	PC-ISO	Pharmaceutical industry, biomedical engineering, food packaging
	Acrylonitrile-butadiene-styrene (ABS)	ABS-M30i	Products in contact with skin, food and medicines
	Polyetheretherketone (PEEK)	LUVOCOM 3F PEEK 9581	Production of surgical and dental instruments
	Polyetherimide (PEI)	ULTEM 1010	Production of surgical and dental instruments
MJ Material Jetting (PolyJet)	Acrylic resins	MED610	Dentistry, orthodontic laboratories in the production of, among other things, crowns and dental bridges
		VeroGlaze (MED620)	
		VeroDent MED670	
		VeroDentPlus MED690	
PBF Selective Laser Sintering (SLS)	Polyamide (PA)	PA 2105	Production of dental instruments
VPP Stereolithography (SLA)	Acrylic resins	dental SG	Production of precise surgical measures and dental models
		dental LT clear	
	Epoxy resin	Accura^®^ ClearVue™	
VPP Digital Light Processing (DLP)	Acrylic resins	3Delta Model 320 3Delta Model Ortho	Production of prosthetic models, gingival masks, surgical templates, orthodontic models

**Table 4:** Techniques for producing polymer–HA composites for use in transitional dental implants.

Polymer–HA Composite	Manufacturing Technique	References
PLA/HA	Vacuum-assisted solvent casting	[187] (2019)
	Extrusion and injection moulding	[301] (2018)
	Fused deposition melting	[302] (2017)
	3D printing	[303] (2016)
	Extrusion process	[304] (2014)
	Electrospinning	[305] (2013)
	Air jet spinning	[306] (2013)
	Stereolithography	[307] (2013)
	Hot pressing	[48] (2006)
	Solvent casting	[308] (2005)
PLLA/HA	Precipitation	[309] (2016)
	Thermally induced phase separation	[310] (2016)
	Solvent casting	[311] (2013)
	Laser melt electrospinning	[312] (2012)
	Melt extrusion	[313] (2010)
	Phase inversion	[314] (2010)
	Freeze extraction	[315] (2010)
	Hot pressing	[316] (2009)
	Electrospinning	[317] (2009)
	Two-step immersing replication	[318] (2008)
	Selective laser sintering	[319] (2005)
PLGA/HA	Solvent casting and injection moulding	[320] (2017)
	Injection moulding	[321] (2015)
	Freeze drying	[322] (2014)
	Solution mixing	[323] (2013)
	Selective laser sintering	[324] (2013)
	Co-solution	[325] (2012)
	Supercritical fluid extractor	[326] (2011)
	Electrospinning	[327] (2011)
	Gas foaming and particulate leaching	[328] (2006)
PCL/HA	Co-extrusion	[329] (2017)
	Freezing of emulsions	[330] (2013)
	Electrospinning	[331] (2010)
	Selective laser sintering	[332] (2010)
	Polymer impregnating	[333] (2008)
	Fuse deposition melting	[334] (2007)
PEEK/HA	Electrophoretic deposition and suspension	[335] (2018)
	Sputtering	[336] (2017)
	Post-deposition heat treatment	[337] (2016)

## Data Availability

Not applicable.
